# Bioinformatic Identification of Neuroblastoma Microenvironment-Associated Biomarkers with Prognostic Value

**DOI:** 10.1155/2020/5943014

**Published:** 2020-09-10

**Authors:** Yi Wang, Huan Luo, Jing Cao, Chao Ma

**Affiliations:** ^1^Department of Neonatology and Neonatal Intensive Care, Zhumadian Central Hospital, Zhumadian, China; ^2^Charité–Universitätsmedizin Berlin, Freie Universität Berlin, Humboldt-Universität zu Berlin, and the Berlin Institute of Health, Berlin, Germany; ^3^Klinik für Augenheilkunde, Charité–Universitätsmedizin Berlin, Freie Universität Berlin, Humboldt-Universität zu Berlin, and Berlin Institute of Health, Berlin, Germany; ^4^Department of Anatomy, College of Basic Medicine, Zhengzhou University, Zhengzhou, China; ^5^BCRT-Berlin Institute of Health Center for Regenerative Therapies, Charité–Universitätsmedizin Berlin, Berlin, Germany

## Abstract

The microenvironment plays a vital role in the tumor recurrence of neuroblastoma. This research aimed at exploring prognostic genes that are involved in neuroblastoma microenvironment. We used “estimate” R package to calculate the immune/stromal/ESTIMATE scores of each sample of ArrayExpress dataset E-MTAB-8248 based on the ESTIMATE algorithm. Then we found that immune/stromal/ESTIMATE scores were not correlated with age/chromosome 11q, but tumor stage, MYCN gene amplifications, and chromosome 1p. Samples were then divided into high- and low-score groups, and 280 common differentially expressed genes (DEGs) were identified. 64 potential prognostic genes were harvested through overall survival analysis from the common DEGs. 14 prognostic genes (ABCA6, SEPP1, SLAMF8, GPR171, ABCA9, ARHGAP15, IL7R, HLA-DPB1, GZMA, GPR183, CCL19, ITK, FGL2, and CD1C) were obtained after screening in two independent cohorts. GO and KEGG analysis discovered that common DEGs and 64 potential prognostic genes are mainly involved in T-cell activation, lymphocyte activation regulation, leukocyte migration, and the interaction of cytokines and cytokine receptors. Correlation analysis showed that all prognostic genes were negatively correlated with MYCN amplification. Cox analysis identified 5 independent prognostic genes (ARHGAP15, ABCA9, CCL19, SLAMF8, and CD1C).

## 1. Introduction

Neuroblastoma is a cancer that develops from immature nerve cells found in multiple parts of the body, including neuroblastomas, ganglioblastomas, and ganglion neuromas [1]. Neuroectodermal cells containing neuroblastomas originate from the neural crest during fetal development and are destined for the adrenal medulla and sympathetic nervous system [1]. Neuroblastoma accounts for 97% of all neuroblastic tumors, is heterogeneous, and differs in location, histopathological appearance, and biological characteristics [1]. Neuroblastoma comprises 6% to 10% of all childhood cancers and causes 15% of all pediatric cancer deaths [2]. Although its molecular basis is still unknown, clinical diversity is closely related to numerous clinical and biological factors, including patient age, tumor stage and histology, and genetic and chromosomal abnormalities [1]. Better learning the molecular mechanism of neuroblastoma could provide a crucial message related to prognosis [3].

The tumor microenvironment is the environment around a tumor, including the surrounding blood vessels, immune cells, fibroblasts, signaling molecules, and the extracellular matrix. The tumor and the surrounding microenvironment are closely related and interact constantly [4]. The tumor microenvironment has been recognized as a complex milieu where cancer cells interact with immune and stromal cells via numerous biochemical and physical signals that are crucial for cancer progression and metastasis [4–6]. The activities of immune cells and stromal cells have been demonstrated owing to the capacity to predict cancer outcomes [7].

ESTIMATE is a method that uses gene expression characteristics to infer the ratio of stromal cells to immune cells in a tumor sample. ESTIMATE scores are correlated with tumor purity based on DNA copy number in samples from 11 different tumor types. These samples have been profiled on Agilent and Affymetrix platforms, or based on RNA sequencing, which can be obtained through The Cancer Genome Atlas. The use of 3,809 transcription profiles provided elsewhere in the public domain further confirmed the accuracy of the prediction. The ESTIMATE method can be used to evaluate the presence of stromal cells and immune cell infiltration in tumor samples using gene expression data [8]. According to the ESTIMATE algorithm, researchers have performed a prognostic assessment and are exploring the genetic changes of many malignant tumors [9–11]. But the immune and stromal scores of neuroblastoma have not yet been elucidated. No research has clearly shown whether the ESTIMATE algorithm can be used to predict the prognosis of neuroblastoma.

To understand the molecular pathogenesis of neuroblastoma involved in the tumor microenvironment, in this work, we used the ESTIMATE algorithm in conjunction with multiple cohorts to explore underlying genetic factors in the tumor microenvironment of neuroblastoma and identify prognostic genes.

## 2. Materials and Methods

### 2.1. ArrayExpress Microarray Data

We searched the ArrayExpress (https://www.ebi.ac.uk/arrayexpress/) database [12] using “neuroblastoma” as the keyword and filtered the results by checking “Homo sapiens” as the “Organism” and selecting “ArrayExpress data only.” In the filtered results, we dug into each detailed description of each dataset to see if they contain survival data. Finally, dataset E-MTAB-8248 that comes from the platform of Agilent-020382 Human Custom Microarray 44k (Feature Number version) was selected for this study. The processed data of the annotation table were obtained from the ArrayExpress website.

### 2.2. Pretreatment of Microarray Data

To begin with, the annotation procedure was performed by using the downloaded annotation table. Then, “estimate” R package was used to figure immune, stromal, and ESTIMATE scores of the dataset by ESTIMATE algorithm [8].

### 2.3. Distributions of Immune/Stromal/ESTIMATE Scores in Different Age/Tumor Stage/MYCN Gene Amplifications/Chromosome 1p/Chromosome 11q/Prognosis

All cases were grouped according to their status of age/tumor stage/MYCN gene amplifications/chromosome 1p/chromosome 11q. Wilcoxon signed-rank test (two groups) or Kruskal–Wallis H test (more than two groups) was utilized to assess the distributions of scores.

### 2.4. Differentially Expressed Gene Analysis

Patients were then stratified according to the median of immune and stromal scores. Differentially expressed genes (DEGs) were explored between high and low immune/stromal score groups using the “limma” R package [13]. The cutoff was set as (1) |log2 (fold-change) | > 1 and (2) false discovery rate (FDR) < 0.05. The heatmaps of DEGs were generated from “pheatmap” R package.

### 2.5. Enrichment Analysis of Common DEGs

The “clusterProfiler” R package was applied to perform functional enrichment analysis of common DEGs, including Gene Ontology (GO) and the Kyoto Encyclopedia of Genes and Genomes (KEGG).

### 2.6. Identification of Potential Prognostic Genes

Kaplan–Meier analysis was conducted by the “survival” R package. The Kaplan–Meier curve illustrated the overall survival difference between low and high expressions of each common DEG gene. This relationship was examined by the log-rank test.

### 2.7. Establishment of the Protein-Protein Interaction (PPI) Network of Potential Prognostic Genes

To better study the potential prognostic genes, we constructed a PPI network using the STRING online database (http://string-db.org) and the Cytoscape software (http://www.cytoscape.org/). The degree distribution was calculated using “cytoHubba” plug-in and Network Analyzer from Cytoscape. The clusters (highly interconnected regions) in this PPI network were discovered by a Cytoscape plug-in named “MCODE.”

### 2.8. Screening for Prognostic Genes in Two Independent Cohorts

Neuroblastoma patients with expression and survival data were available in the datasets of GSE85047 and GSE49710 from the Gene Expression Omnibus (GEO, https://www.ncbi.nlm.nih.gov/geo/) database. The gene expression profiles were measured experimentally using platforms of GPL5175 and GPL16876, respectively (Table 1). We screened the identified potential prognostic genes using the two cohorts by overall survival. *p* value <0.05 (log-rank test) was considered statistically significant. The genes that are in the intersection of the above two results were considered prognostic genes.

### 2.9. Identification of the Independent Prognostic Value of Prognostic Genes

MYCN amplification was strongly correlated with a poor prognosis in neuroblastoma cases [14]. The status of MYCN and the expression of prognostic genes were extracted from GSE49710, and their correlation was checked using the Spearman test. In addition, to explore the independence of the prognostic genes, we performed univariate and multivariate Cox analysis on prognostic genes and MYCN in the GSE49710 cohort. In the end, we reviewed previous studies related to crucial prognostic genes and neuroblastoma.

## 3. Results

### 3.1. Characteristics of Cohorts Included in This Study

E-MTAB-8248 (*n* = 223) was chosen for identifying potential prognostic genes in the TME of neuroblastoma. GSE85047 (*n* = 283) and GSE49710 (*n* = 498) were taken as screening tools to obtain prognostic genes from potential genes. The clinical characteristics of the cohorts included in this study are shown in Table 2.

### 3.2. Distributions of Immune/Stromal/ESTIMATE Scores in Different Age/Tumor Stage/MYCN Gene Amplifications/Chromosome 1p/Chromosome 11q/Prognosis

**Figure 1:**
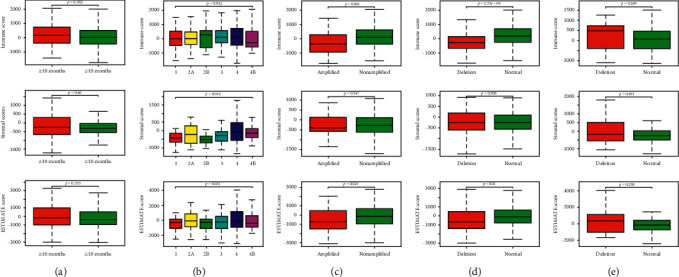
Correlations between immune/stromal/ESTIMATE scores and age/tumor stage/MYCN gene amplifications/chromosome 1p/chromosome 11q. Distribution of immune scores (upper), stromal scores (middle), and ESTIMATE scores (bottom) plotted against age (a), tumor stage (b), MYCN gene amplifications (c), chromosome 1p (d), and chromosome 11q (e). *p* value <0.05 was considered statistically significant.

The distributions of immune/stromal/ESTIMATE scores did not vary with age/chromosome 11q (Figure 1). However, in the tumor stage section, the distribution of stromal scores was significantly different in the stages, but immune and ESTIMATE scores not. In the distribution of scores of MYCN gene amplifications, immune and ESTIMATE scores mattered, but stromal score not. The same happened in the distribution of chromosome 1p scores, the stromal score could not differentiate the status of chromosome 1p, but immune and ESTIMATE scores could.

### 3.3. Immune/Stromal/ESTIMATE Scores and Prognosis

**Figure 2:**
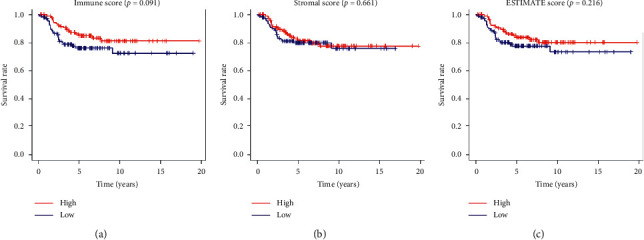
Association of immune/stromal/ESTIMATE scores with overall survival. High (red) and low (blue) immune (a)/stromal (b)/ESTIMATE (c) scores have correlations with prognosis.

223 neuroblastoma patients were divided into high- and low-score groups according to their median scores. Kaplan–Meier survival curves showed that high immune score patients had a better trend of overall survival than that the low-score group (*p* value = 0.091 in the log-rank test) (Figure 2(a)). Analogously, as shown in Figures 2(b) and 2(c), patients with high stromal/ESTIMATE scores achieved a better trend on overall outcomes than those with low scores (*p* value >0.216). Not noticeable significant statistical results simply predict that fewer microenvironment-related genes may be found in the next steps and do not negate the role of the microenvironment in neuroblastoma. So, we still will focus on the genes related to both the immune and stromal scores.

### 3.4. Identification of Common DEGs from Immune and Stromal Scores

**Figure 3:**
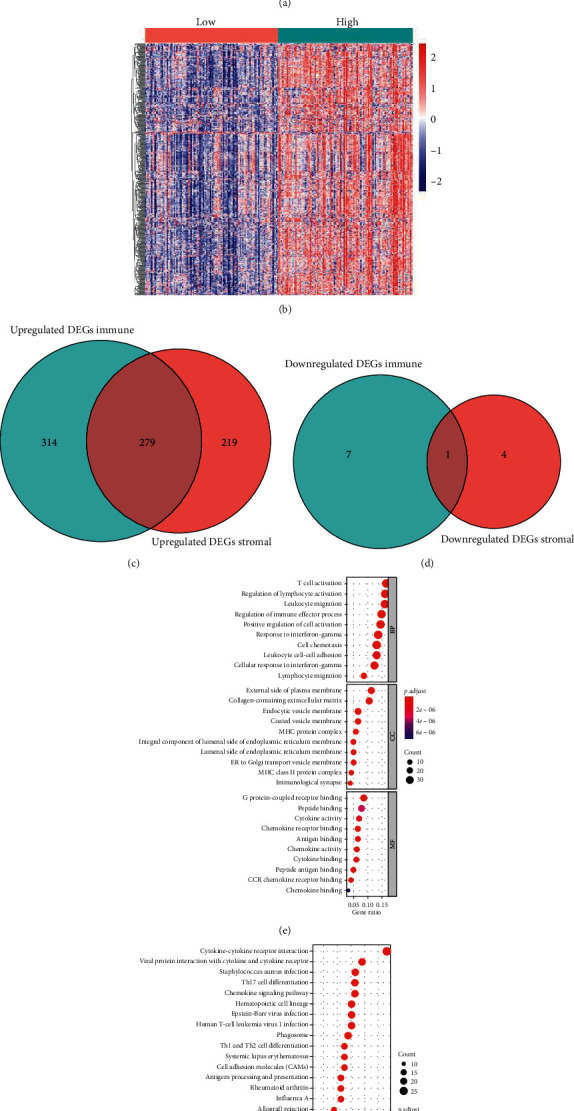
Identification of common DEGs from immune and stromal scores in neuroblastoma. (a) Heatmap of the DEGs of immune scores (cutoff: |log_2_(fold-change) |>1, FDR < 0.05). (b) Heatmap of the DEGs of stromal scores (cutoff: |log2 (fold-change) |>1, FDR < 0.05). (c, d) Venn diagrams of commonly upregulated (c) and downregulated (d) DEGs in stromal and immune score groups. (e, f) Top ten GO terms (e) and top thirty KEGG pathway (f) enrichment of common DEGs (FDR < 0.05). DEG: differential gene expression; GO: gene ontology; BP: biological process; CC: cellular component; MF: molecular function; KEGG: Kyoto Encyclopedia of Genes and Genomes; FDR: false discovery rate.

223 neuroblastoma patients were grouped based on their median scores. As shown in Figure 3(a), 601 DEGs were found among immune score groups (Table S1). 503 DEGs among stromal score groups were discovered, which are shown in Figure 3(b) and Table S2. 279 common upregulated DEGs (Figure 3(c)) and 1 common downregulated DEG (Figure 3(d)) were identified then by comprehensive analysis. The 280 common DEGs (Table S3) then entered into the next steps.

GO enrichment analysis (Table S4) showed top GO terms identified in the 280 common DEGs mainly involved with lymphocyte differentiation, T-cell activation, regulation of lymphocyte activation, external side of the plasma membrane, collagen-containing extracellular matrix, G protein-coupled receptor binding, and the peptide binding (Figure 3(e)). KEGG analysis (Figure 3(f) and Table S5) of 280 common DEGs was mainly observed for cytokine-cytokine receptor interaction, viral protein interaction with cytokine and cytokine receptor, *Staphylococcus aureus* infection, Th17 cell differentiation, and chemokine signaling pathway.

### 3.5. Identification of Potential Prognostic Genes

**Figure 4:**
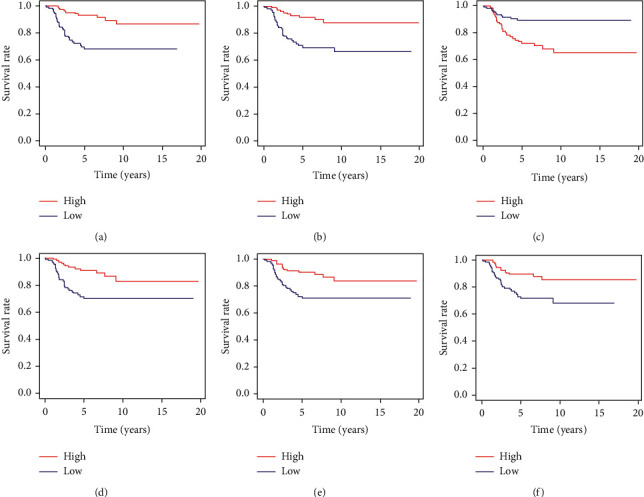
Representative Kaplan–Meier curves of potential prognostic genes based on overall survival in the ArrayExpress dataset E-MTAB-8248. *p* value <0.05 was used to assess differences in the log-rank test. (a) RGS1 (*p* < 0.001). (b) CXCR6 (*p* < 0.001). (c) CYP1B1 (*p* < 0.001). (d) IL7R (*p* = 0.001). (e) PTGDS (*p* = 0.001). (f) SMOC2 (*p* = 0.004).

Kaplan–Meier survival curves were built for the 280 common DEGs based on overall survival. According to the log-rank test (*p* value <0.05), 64 common DEGs had survival predictive ability (all were upregulated in TME), of which 60 and 4 were positively and negatively correlated with survival, respectively (Figure 4 and Table S3).

### 3.6. Functional Enrichment Analysis and Protein-Protein Interaction Construction of Potential Prognostic Genes

**Figure 5:**
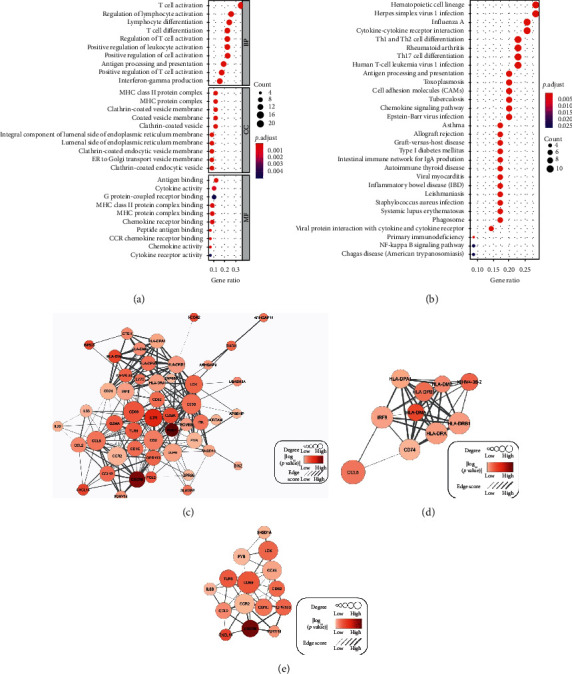
Identification of the enrichments of GO and KEGG of the potential prognostic genes and PPI network construction. (a) Top ten GO terms (FDR < 0.05). (b) Top thirty KEGG pathways (FDR < 0.05). (c, d, e) PPI network (c) with two clusters: cluster one and cluster two (d, e). The *p* values shown in (c, d, e) were harvested from the overall survival analysis on the ArrayExpress dataset E-MTAB-8248. “Degree” was generated using the “cytoHubba” plugin of Cytoscape software (http://www.cytoscape.org/). “Combine score” means the strength between two-gene, and it was from STRING online database (http://string-db.org). GO: gene ontology; BP: biological process; CC: cellular component; MF: molecular function; KEGG: Kyoto Encyclopedia of Genes and Genomes; FDR: false discovery rate; PPI: protein-protein interaction; Edge score: combine score.

GO enrichment was conducted using the 64 prognostic genes, and the enriched items in GO were involved largely in T-cell activation and the regulation of lymphocyte activation, MHC class II protein complex and the MHC protein complex, and antigen binding and the cytokine activity (Figure 5(a)). KEGG pathways were mainly enriched for hematopoietic cell lineage, herpes simplex virus 1 infection, influenza A, and cytokine-cytokine receptor interaction (Figure 5(b)).

A PPI network was constructed by the online STRING tool and Cytoscape software. After hiding disconnected nodes, there were 50 nodes and 239 edges in this network (Figure 5(c)). All genes positively correlated with the overall outcomes of neuroblastoma cases. CD69, CD3D, CD2, CCL5, CCR2, CD48, LCK, IL7R, IRF8, and HLA-DRB1 were the top ten genes in this network sorted by degree. RGS1 had the strongest predictive ability in this network for overall survival; however, FYB had the weakest ability to do so. The interaction between HLA-DRA and CD74 was the tightest in this PPI network, with a combine score of 0.999. The loosest relationship happened between CD52 and CCR2, with a combine score of 0.409.

Then, we explored the PPI network using the Cytoscape “MCODE” plug-in and found 3 main clusters. The top two modules had more than 9 nodes, which were plotted by us (Figures 5(d) and 5(e)). In cluster one, CCL5, HLA-DRA, HLA-DRB1, IRF8, and CD74 were the top five genes sorted by degree, and HLA-DMA, IGHV4-38-2, HLA-DPB1, CCL5, and HLA-DMB were the top five genes sorted by prognostic ability. In cluster two, CD69, CCR2, CD48, LCK, and TLR8 were on the top five sorted by degree distribution, and CXCR6, CXCL14, CD69, TLR8, and GPR183 were on the top five sorted by prognostic ability.

### 3.7. Screening in Two GEO Cohorts

We then screened the 64 potential prognostic genes described above using the GSE85047 and GSE49710 cohorts from the GEO database. *p* value <0.05 in the log-rank test was set as the cutoff. 14 genes were screened from GSE85047 cohort (Table S6), 56 genes were found from GSE49710 (Table S7), and 14 in the intersection were identified as prognostic genes (Table 1).

### 3.8. Identification of the Independent Prognostic Value of Prognostic Genes

MYCN amplification was strongly correlated with a poor prognosis in neuroblastoma cases [14]. The status of MYCN and the expression of prognostic genes were extracted from GSE49710, and their correlation was checked using the Spearman test. The correlation results shown in Table 3 indicated that all prognostic genes were negatively correlated with the amplification of MYCN. In univariate Cox analysis, all prognostic genes and MYCN amplification were related to prognosis. In multivariate Cox analysis, MYCN amplification, ARHGAP15, ABCA9, CCL19, SLAMF8, and CD1C could still predict prognosis (Table 4). The above results suggested that ARHGAP15, ABCA9, CCL19, SLAMF8, and CD1C were potential independent prognostic factors for neuroblastoma. Finally, we did a literature review, finding that, among 14 prognostic genes identified in the present study, 3 had been reported having experimental evidence involving in the progression of neuroblastoma, none of them had been previously experimentally proofed affecting neuroblastoma prognosis (Table 5).

## 4. Discussion

**Figure 6:**
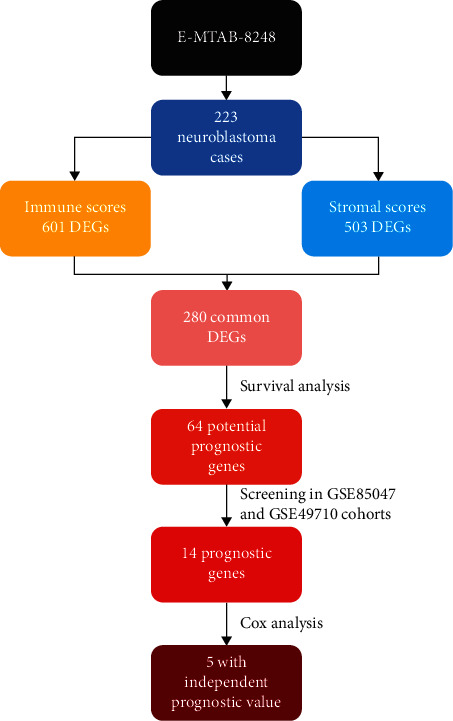
The workflow of the present study. Dataset E-MTAB-8248 was from ArrayExpress database (https://www.ebi.ac.uk/arrayexpress/); Datasets GSE85047 and GSE49710 were from Gene Expression Omnibus database (https://www.ncbi.nlm.nih.gov/geo/); DEG: differentially expressed gene.

In this study, we attempted to identify tumor microenvironment-related genes that contribute to neuroblastoma outcome from an ArrayExpress dataset. By comparing global gene expression between high- and low-score patients, 280 common DEGs were found. Then 64 genes were identified owning potential prognostic value via survival analysis. Importantly, we screened the 64 genes in two independent GEO cohorts, identifying 14 genes (ABCA6, SEPP1, SLAMF8, GPR171, ABCA9, ARHGAP15, IL7R, HLA-DPB1, GZMA, GPR183, CCL19, ITK, FGL2, and CD1C) as prognostic genes. Moreover, ARHGAP15, ABCA9, CCL19, SLAMF8, and CD1C were found via Cox analysis having independent prognostic value (Figure 6).

We deployed the ESTIMATE algorithm to figure the immune/stromal/ESTIMATE scores of neuroblastoma cases and found these scores were not correlated with age/chromosome 11q, but tumor stage, MYCN gene amplifications, and chromosome 1p. The MYCN, a member of the MYC family, has been associated with high-risk disease and poor prognosis in approximately 25% of cases of neuroblastoma. Currently, the amplification of MYCN is still one of the most risky genetic markers in neuroblastoma [18]. Neuroblastoma tumor cells lacking parts of chromosome 1 or 11 (known as 1p deletion or 11q deletion) may be predictive of poor prognosis [19]. In our study, we found that the scores were related to the MYCN gene amplifications and chromosome 1p, which may reveal a new understanding of this disease, but need further research. And we also found the scores were related to the prognosis of neuroblastoma. Immune cells and stromal cells are essential components active in the tumor microenvironment, which affect the survival, proliferation, and treatment resistance of neuroblastoma [20–24]. The crosstalk between tumor and microenvironment influences the inflammatory response: cancer cells interact with both the innate and the adaptive immune system and use immune cells for tumor survival and protection from immunological attacks [21, 24–26].

GO analysis showed the common DEGs were largely enriched in T-cell activation, external side of the plasma membrane, and G protein-coupled receptor binding. Besides, the KEGG analysis demonstrated that cytokine-cytokine receptor interaction, viral protein interaction with cytokine and cytokine receptor, and *Staphylococcus aureus* infection were mostly enriched. Previous studies have shown that the biological processes of the immune system are critical to the formation of a complex tumor microenvironment, which is consistent with our findings [21, 22, 24]. In the last few years, the immunological characteristics of neuroblastoma have been more deeply understood, and the development of effective neuroblastoma immunotherapy strategies has attracted widespread attention [27–29].

Overall survival analysis of the common DEGs found 64 genes owned potential prognosis capacity in neuroblastoma cases. In the PPI network of these 64 potential prognostic genes, we are particularly interested in CCL5 in cluster one (Figure 5(d)) and CD69 and TLR8 in cluster two (Figure 5(e)), of whom hold the top five degree and prognostic ability in their own cluster. CCL5 is an inflammatory chemokine that is widely secreted from natural killer cells, T cells, fibroblasts, epithelial cells, and platelets [30] and promotes chemotaxis on cells involved in the immune/inflammatory response [31]. Studies demonstrated that CCL5 is related to certain tumor cells, such as malignant melanoma cells [32], ovarian [33], prostate [34], and breast cancer cells [35]. The exact functions of CCL5 in tumor biology are still unclear. CCL5 production is relevant to inducing proper immune responses against tumors [36]. The current research showed that CCL5 promotes tumor migration and invasion. CCL5 secreted by tumor cells and other cells in the breast cancer tumor microenvironment can recruit tumor-associated macrophages into the tumor microenvironment [31]. Evidence on the direct relationship between CCL5 and neuroblastoma is still lacking and requires further study. CD69, a type II glycoprotein known to regulate inflammation through T-cell migration and retention in tissues, plays an important role in inducing the exhaustion of tumor-infiltrating T cells [37]. CD69 expression is readily upregulated upon activation in most leukocytes, which mainly underlies its widespread use as a marker of activated lymphocytes and NK cells [38]. Mita et al. reported that the use of anti-CD69 monoclonal antibodies to treat tumor-bearing mice significantly reduced tumor-infiltrating T-cell failure and enhanced protection against metastasis, indicating the efficacy of anti-CD69 antibodies in the treatment of malignant tumors [37]. CD69 expression is associated with hypoxia in the tumor microenvironment [39]. The research on the relationship between CD69 and tumors is still scarce, is still in infancy, and needs more in-depth research. TLR7 and TLR8 are phylogenetically and structurally closely related members of the TLR family; together with TLR9, they constitute one of the six major TLR clades [40]. TLR8 has been identified as a natural receptor for single-stranded RNA and is thought to act as an effective activator of the innate immune response after viral infection [41–43]. TLR8 is the only TLR that has been shown to be necessary and sufficient to reverse the suppressive function of Treg cells, resulting in strong tumor-suppressive effects [44]. Numerous reports have described the Toll-like receptor (TLR) expression in the tumor microenvironment as it relates to cancer progression, as well as their involvement in inflammation [45]. Inflammation triggered by TLR signaling can directly influence tumor-induced senescence in tumor microenvironments, and the effects are variable depending on different TLR signaling and tumor types [46].

In this study, GSE85047 and GSE49710 cohorts were used for the screening tools for prognostic genes. We screened the prognostic value of these 64 genes based on the cohorts, of which 14 genes were identified as prognostic genes. ARHGAP15, ABCA9, CCL19, SLAMF8, and CD1C could predict prognosis independently. We then conducted a small-scale review of these 14 genes, finding IL7R, CCL19, and GZMA have been demonstrated having experimental evidence with the progression of neuroblastoma by the previous research [15–17], while no gene has been experimentally confirmed having roles in prognosis to neuroblastoma. Several gene expression studies indicate that IL7 has increased expression in NB specifically in tumors with a better prognosis, making IL7 a key candidate for this soluble factor. Prasad's finding implicated IL7 within the Schwannian stroma of the neuroblastoma tumor architecture as having a paracrine signaling effect on neighboring neuroblasts which may provide the antiproliferative and differentiation signals postulated [15]. Walker and colleagues found that changes in the CCL19 signal transduction pathway can lead to defects in the migration of dendritic cells in neuroblastoma, which in turn affects tumor progression [16]. Yarmarkovich demonstrated that GZMA has a strong correlation with T-cell factors, and GZMA is very active in the neuroblastoma tumor microenvironment [17]. Most of the prognostic genes obtained in our study have not been clarified the mechanism in neuroblastoma, and more efforts need to be implemented in the future.

## 5. Conclusion

In summary, by applying ESTIMATE algorithm, and mining from ArrayExpress dataset and other two independent GEO cohorts, we got 14 prognostic genes (ABCA6, SEPP1, SLAMF8, GPR171, ABCA9, ARHGAP15, IL7R, HLA-DPB1, GZMA, GPR183, CCL19, ITK, FGL2, and CD1C) having the capacity to illustrate the prognosis of neuroblastoma patients. Besides, ARHGAP15, ABCA9, CCL19, SLAMF8, and CD1C had independent prognostic value. Interestingly, only 3 of the 14 genes have been previously experimentally identified to be involved in the progression of neuroblastoma. It would be anticipated to test whether this set of genes can provide better survival prediction capabilities than individual genes. More efforts on the further research of the 14 genes may reveal a potential relationship among tumor microenvironment and neuroblastoma prognosis in a novel and comprehensive means.

## Figures and Tables

**Table 1:** Genes identified in this study as prognostic markers.

Gene	Kaplan–Meier analysis (*p* value)			Prognostic correlation	Regulation in TME
	E-MTAB-8248	GSE85047	GSE49710		
ABCA6	0.048329644	0.000456	1.38*E* − 11	Positively	Upregulated
SEPP1	0.043655597	0.000644	4.85*E* − 05	Positively	Upregulated
SLAMF8	0.016798116	0.00237	1.44*E* − 09	Positively	Upregulated
GPR171	0.013243245	0.006901	3.27*E* − 07	Positively	Upregulated
ABCA9	0.04278403	0.007713	5.43*E* − 09	Positively	Upregulated
ARHGAP15	0.013917259	0.010945	1.6*E* − 05	Positively	Upregulated
IL7R	0.001138517	0.012055	1.44*E* − 07	Positively	Upregulated
HLA-DPB1	0.014579249	0.020717	2.25*E* − 05	Positively	Upregulated
GZMA	0.00900038	0.023614	0.000124	Positively	Upregulated
GPR183	0.010691506	0.024086	4.64*E* − 06	Positively	Upregulated
CCL19	0.013557588	0.034966	8.77*E* − 07	Positively	Upregulated
ITK	0.016816901	0.044203	2.78*E* − 06	Positively	Upregulated
FGL2	0.007567598	0.045289	2*E* − 06	Positively	Upregulated
CD1C	0.013928199	0.047954	2.7*E* − 09	Positively	Upregulated

TME: the tumor microenvironment.

**Table 2:** Clinical characteristics of cohorts involved in the study.

Characteristics	E-MTAB-8248, *n* = 223	GSE85047, *n* = 283	GSE49710, *n* = 498
Age (months)			
≤18	103 (46.19%)	144 (50.88%)	300 (60.24%)
>18	120 (53.81%)	134 (47.35%)	198 (39.76%)
Unknown	0	5 (1.77%)	0
Gender			
Female	NA	NA	211 (42.37%)
Male	NA	NA	287 (57.63%)
MYCN			
Amplified	46 (20.63%)	55 (19.43%)	92 (18.47%)
Nonamplified	176 (78.92%)	222 (78.45%)	401 (80.52%)
Unknown	1 (0.45%)	6 (2.12%)	5 (1%)
Stage (INSS)			
1	29 (13%)	50 (17.67%)	121 (24.3%)
2	39 (17.49%)	36 (12.72%)	78 (15.66%)
3	36 (16.14%)	43 (15.19%)	63 (12.65%)
4	89 (39.91%)	124 (43.82%)	183 (36.75%)
4s	30 (13.45%)	27 (9.54%)	53 (10.64%)
Unknown	0	3 (1.06%)	0
1p status			
Del/im	67 (30.04%)	NA	NA
Normal	137 (61.43%)	NA	NA
Unknown	19 (8.52%)	NA	NA
11q status			
Del	18 (8.07%)	NA	NA
Normal	46 (20.63%)	NA	NA
Unknown	159 (71.3%)	NA	NA

**Table 3:** The correlations between prognostic genes and the status of MYCN status tested by Spearman correlation.

Gene	*R*	*p* value
ABCA6	−0.433035927	5.96*E* − 24
SEPP1	−0.43773706	1.71*E* − 24
SLAMF8	−0.485479	1.62*E* − 30
GPR171	−0.449936802	6.05*E* − 26
ABCA9	−0.371500185	1.40*E* − 17
ARHGAP15	−0.452056568	3.34*E* − 26
IL7R	−0.457452749	7.23*E* − 27
HLA-DPB1	−0.418455553	2.55*E* − 22
GZMA	−0.411284919	1.51*E* − 21
GPR183	−0.415347624	5.55*E* − 22
CCL19	−0.462592772	1.64*E* − 27
ITK	−0.483115504	3.40*E* − 30
FGL2	−0.459540146	3.97*E* − 27
CD1C	−0.466033388	5.99*E* − 28

**Table 4:** The Cox analysis on prognostic genes and MYCN in the GSE49710 cohort.

Variable	Univariate Cox analysis				Multivariate Cox analysis^*∗*^			
	Coef	HR (95% CI)	*z*	*p* value	Coef	HR (95% CI)	*z*	*p* value
ABCA6	−0.345	0.708 (0.651–0.77)	−8.1	5.38*E* − 16	0.0186	1.02 (0.776–1.34)	0.134	0.894
SEPP1	−0.392	0.676 (0.602–0.759)	−6.64	3.11*E* − 11	−0.0928	0.911 (0.644–1.29)	−0.524	0.6
SLAMF8	−0.505	0.603 (0.537–0.677)	−8.54	1.29*E* − 17	−0.274	0.76 (0.586–0.986)	−2.07	0.0389
GPR171	−0.415	0.66 (0.582–0.749)	−6.44	1.21*E* − 10	−0.108	0.898 (0.581–1.39)	−0.484	0.629
ABCA9	−0.387	0.679 (0.613–0.753)	−7.34	2.10*E* − 13	−0.303	0.738 (0.574–0.95)	−2.36	0.0183
ARHGAP15	−0.393	0.675 (0.598–0.763)	−6.31	2.77*E* − 10	0.583	1.79 (1.22–2.62)	3	0.00272
IL7R	−0.375	0.687 (0.617–0.765)	−6.86	7.05*E* − 12	−0.112	0.894 (0.624–1.28)	−0.607	0.544
HLA-DPB1	−0.403	0.668 (0.588–0.759)	−6.21	5.40*E* − 10	0.0422	1.04 (0.735–1.48)	0.236	0.813
GZMA	−0.305	0.737 (0.666–0.816)	−5.86	4.62*E* − 09	0.216	1.24 (0.993–1.55)	1.89	0.0581
GPR183	−0.387	0.679 (0.604–0.763)	−6.51	7.63*E* − 11	−0.0716	0.931 (0.746–1.16)	−0.633	0.527
CCL19	−0.184	0.832 (0.783–0.884)	−5.94	2.81*E* − 09	0.147	1.16 (1.01–1.33)	2.09	0.0362
ITK	−0.35	0.705 (0.641–0.775)	−7.25	4.04*E* − 13	−0.125	0.883 (0.662–1.18)	−0.85	0.395
FGL2	−0.383	0.682 (0.612–0.76)	−6.95	3.66*E* − 12	0.0172	1.02 (0.677–1.53)	0.0827	0.934
CD1C	−0.356	0.701 (0.64–0.767)	−7.68	1.65*E* − 14	−0.235	0.791 (0.633–0.989)	−2.06	0.0393
MYCN amplification	2.05	7.8 (5.26–11.5)	10.2	1.19*E* − 24	1.7	5.45 (3.22–9.21)	6.33	2.48*E* − 10

^*∗*^Concordance = 0.809 (se = 0.022), likelihood ratio test = 129.7 on 15 df, *p* ≤ 2*e* − 16; Wald test = 139.2 on 15 df, *p* ≤ 2*e* – 16; score (log-rank) test = 186.3 on 15 df, *p* ≤ 2*e* − 16.

**Table 5:** Prognostic genes identified related to the tumor microenvironment in neuroblastoma.

Categories^#^	Gene symbols^$^
Molecular function	ABCA6, ABCA9, **IL7R** [15], GPR183, **CCL19** [16]
Extracellular region	SEPP1, **GZMA** [17], FGL2
Plasma membrane	GPR171, HLA-DPB1, CD1C
Signal transduction	ARHGAP15, ITK
Membrane	SLAMF8

^#^Classified by gene ontology terms. ^$^Genes in bold were previously reported having experimental evidence involving in the progression of neuroblastoma.

## Data Availability

Publicly available datasets were analyzed in this study. These data can be found as follows: ArrayExpress: https://www.ebi.ac.uk/arrayexpress/, and GEO: https://www.ncbi.nlm.nih.gov/geo/.

## Supplementary Materials

Supplementary Materials Table S1: 601 DEGs found between immune score groups (FDR < 0.05). Table S2: 503 DEGs found between stromal score groups (FDR < 0.05). Table S3: the performance of 280 common DEGs in Kaplan–Meier analysis from the E-MTAB-8248 cohort with *p* value tested in the log-rank test. Table S4: the gene ontology term enrichment of 280 common differentially expressed genes with *p*.adjust <0.05. Table S5: the Kyoto Encyclopedia of Genes and Genomes pathway enrichment of 280 common differentially expressed genes with *p*.adjust <0.05. Table S6: the list of 14 prognostic genes screened from the GSE85047 cohort with *p* value <0.05 in the log-rank test. Table S7: the list of 56 prognostic genes screened from the GSE49710 cohort with *p* value < 0.05 in the log-rank test. Table S8: the main scripts used in this study.
