# Supplementary material for: Bioinformatic Identification of Neuroblastoma Microenvironment-Associated Biomarkers with Prognostic Value
Source: J Oncol. 2020 Sep 10;2020:5943014. doi: 10.1155/2020/5943014 (PMC7501561; doi:10.1155/2020/5943014)
Supplement: Supplementary Materials — Table S1: 601 DEGs found between immune score groups (FDR < 0.05). Table S2: 503 DEGs found between stromal score groups (FDR < 0.05). Table S3: the performance of 280 common DEGs in Kaplan–Meier analysis from the E-MTAB-8248 cohort with p value tested in the log-rank test. Table S4: the gene ontology term enrichment of 280 common differentially expressed genes with p.adjust <0.05. Table S5: the Kyoto Encyclopedia of Genes and Genomes pathway enrichment of 280 common differentially expressed genes with p.adjust <0.05. Table S6: the list of 14 prognostic genes screened from the GSE85047 cohort with p value <0.05 in the log-rank test. Table S7: the list of 56 prognostic genes screened from the GSE49710 cohort with p value < 0.05 in the log-rank test. Table S8: the main scripts used in this study. [file 5943014.f1.pdf]

Table S1. 601 DEGs found between immune score groups (FDR<0.05).

gene	logFC	pvalue	FDR
ABCA12	-1.07535	0.003423	0.013284
ABCA6	1.405363	8.39E-15	2.70E-13
ABCA9	1.164557	5.03E-10	9.36E-09
ABCC3	1.11528	3.72E-13	9.88E-12
ACP5	1.123026	1.57E-12	3.93E-11
ACSL5	1.189027	5.36E-25	9.20E-23
ADA	1.14838	1.72E-21	1.38E-19
ADAM28	1.318904	1.01E-18	5.39E-17
ADAMDEC1	1.509033	5.94E-16	2.21E-14
ADH1A	1.209836	1.08E-07	1.47E-06
AHR	1.239921	1.17E-20	8.24E-19
AIF1	1.410302	2.43E-29	2.68E-26
AIM1	1.456339	3.87E-27	1.32E-24
AIM2	1.210624	1.12E-15	4.02E-14
ALDH1A1	1.161957	3.42E-10	6.50E-09
ALOX5	1.382257	1.41E-21	1.15E-19
ALOX5AP	1.533584	3.72E-22	3.42E-20
AMICA1	2.00393	8.83E-28	4.34E-25
ANKRD22	1.393395	1.55E-13	4.31E-12
ANKRD55	1.042877	3.14E-08	4.61E-07
AOAH	1.486373	4.10E-24	5.72E-22
APBB1IP	1.498871	3.39E-30	6.00E-27
APOB48R	1.122665	4.11E-22	3.75E-20
APOBEC3G	1.050973	1.36E-15	4.79E-14
APOBEC3H	1.581975	2.30E-19	1.37E-17
APOC1	1.00898	2.19E-12	5.41E-11
APOD	1.436204	8.62E-08	1.19E-06
APOL3	1.364254	7.41E-25	1.25E-22
ARHGAP15	1.412461	3.73E-21	2.85E-19
ARHGAP9	1.766045	1.36E-31	4.82E-28
ARHGDIB	1.270451	2.35E-27	8.86E-25
ARID5A	1.059517	4.64E-20	3.06E-18
ARRDC2	1.160373	3.72E-22	3.42E-20
ARRDC5	1.344028	1.38E-16	5.58E-15
ASB2	1.131966	1.28E-15	4.53E-14
ATHL1	1.362421	9.93E-19	5.34E-17
B2M	1.161721	5.99E-21	4.38E-19
BANK1	1.751144	1.25E-14	3.93E-13
BATF	1.556404	2.51E-25	4.72E-23
BCL3	1.317777	2.23E-22	2.20E-20
BIC	1.119735	1.34E-15	4.72E-14
BIN2	1.5672	6.41E-28	3.34E-25

BIRC3	2.274251	3.33E-25	6.07E-23
BLK	1.513146	4.64E-14	1.37E-12
BLNK	1.534848	1.65E-18	8.50E-17
BST2	1.213587	6.19E-17	2.65E-15
BTK	1.250745	5.41E-23	5.70E-21
BTLA	1.5804	1.94E-17	8.95E-16
BTN3A2	1.255278	7.24E-19	3.99E-17
BTN3A3	1.111986	2.11E-15	7.22E-14
C10orf116	1.158496	4.71E-10	8.80E-09
C10orf56	1.023275	1.75E-11	3.83E-10
C12orf42	1.031592	1.48E-10	2.96E-09
C13orf18	1.208099	1.13E-13	3.20E-12
C14orf56	1.080762	2.40E-09	4.11E-08
C17orf60	1.107109	6.19E-16	2.30E-14
C1QA	1.277175	2.64E-21	2.06E-19
C1QB	1.090693	6.30E-17	2.69E-15
C1QC	1.130934	7.51E-17	3.16E-15
C1R	1.740221	1.37E-23	1.65E-21
C1S	1.942737	2.56E-23	2.90E-21
C1orf179	1.059622	1.16E-08	1.81E-07
C1orf38	1.113941	5.08E-23	5.39E-21
C20orf174	1.905954	2.38E-17	1.07E-15
C21orf96	1.420931	3.24E-20	2.16E-18
C3	1.803144	1.17E-17	5.53E-16
C4orf7	3.388739	1.46E-13	4.06E-12
C5orf29	1.215253	2.43E-16	9.50E-15
C5orf39	1.172193	1.42E-16	5.72E-15
C6orf105	1.017715	3.76E-10	7.10E-09
C6orf32	1.324765	8.93E-13	2.29E-11
C7	1.455089	7.27E-11	1.50E-09
CALB1	-1.30686	0.000145	0.000994
CAPG	1.204179	1.25E-14	3.93E-13
CASP1	1.377402	1.47E-28	1.08E-25
CASP4	1.234711	2.79E-25	5.15E-23
CASP5	1.131877	2.77E-24	3.95E-22
CCDC80	1.033386	5.54E-09	9.01E-08
CCDC88	1.051921	1.56E-15	5.44E-14
CCL11	1.686602	8.58E-12	1.97E-10
CCL13	1.488419	2.94E-15	9.89E-14
CCL14	1.372151	2.69E-11	5.81E-10
CCL18	1.612813	1.58E-11	3.49E-10
CCL19	3.362158	4.89E-19	2.77E-17
CCL2	1.707626	6.94E-20	4.45E-18
CCL21	1.668884	4.91E-14	1.44E-12

CCL5	2.05887	1.31E-29	1.79E-26
CCL8	1.234985	4.54E-09	7.47E-08
CCR2	1.716886	2.18E-20	1.48E-18
CCR5	1.406125	1.11E-23	1.36E-21
CCR6	1.106457	6.63E-09	1.07E-07
CCR7	2.557256	5.47E-19	3.07E-17
CCRL2	1.084157	4.59E-21	3.41E-19
CD160	1.37491	7.30E-14	2.10E-12
CD163	1.15848	9.22E-12	2.11E-10
CD180	1.195069	9.48E-15	3.02E-13
CD19	1.776935	3.27E-14	9.79E-13
CD1B	1.00139	7.64E-08	1.07E-06
CD1C	1.791099	2.66E-21	2.08E-19
CD1E	1.703708	3.30E-15	1.11E-13
CD2	2.244339	1.07E-27	4.87E-25
CD247	1.849091	9.36E-24	1.19E-21
CD27	1.460915	6.29E-22	5.48E-20
CD28	1.707674	3.13E-17	1.39E-15
CD33	1.11199	2.61E-21	2.05E-19
CD37	1.884773	1.27E-27	5.36E-25
CD38	1.661336	7.68E-26	1.66E-23
CD3D	2.412723	1.55E-25	3.05E-23
CD3G	2.041999	2.52E-22	2.44E-20
CD48	2.05283	1.59E-29	2.01E-26
CD52	2.242872	6.50E-27	2.09E-24
CD53	1.764663	2.80E-28	1.77E-25
CD5L	1.054842	0.00033	0.001963
CD6	1.052664	1.97E-21	1.57E-19
CD68	1.103375	1.04E-17	4.95E-16
CD69	2.343019	6.54E-24	8.70E-22
CD72	1.412919	1.78E-22	1.77E-20
CD74	1.752006	8.34E-24	1.08E-21
CD79B	1.491418	3.90E-15	1.29E-13
CD82	1.002912	1.84E-22	1.82E-20
CD84	1.010126	8.72E-19	4.75E-17
CD8B	1.805789	2.65E-20	1.78E-18
CD96	1.827562	6.16E-26	1.35E-23
CD97	1.135944	4.83E-26	1.07E-23
CEBPD	1.026972	5.82E-11	1.21E-09
CENTD1	1.493262	3.66E-29	3.60E-26
CETP	1.220767	8.41E-14	2.40E-12
CFB	1.329729	7.35E-20	4.70E-18
CFD	1.119699	3.19E-14	9.59E-13
CFHR3	1.174706	6.20E-13	1.62E-11

CFI	1.152307	2.04E-15	7.01E-14
CFP	1.103117	6.46E-18	3.11E-16
CH25H	1.171509	2.55E-10	4.92E-09
CHI3L1	1.337132	1.71E-11	3.76E-10
CHI3L2	2.274293	7.78E-17	3.25E-15
CIITA	1.061795	1.56E-26	4.37E-24
CLEC10A	1.111346	7.12E-24	9.33E-22
CLEC2B	1.259998	5.31E-27	1.74E-24
CLEC4D	1.23847	3.61E-10	6.84E-09
CLEC4E	2.019525	1.13E-12	2.85E-11
CLEC4G	1.185055	1.58E-05	0.000143
CLIC2	1.175324	1.38E-22	1.40E-20
CLIC3	1.54103	1.13E-14	3.60E-13
CLIC5	1.08553	8.18E-15	2.64E-13
CLIC6	1.240657	4.68E-09	7.69E-08
CMTM7	1.153831	1.22E-24	1.87E-22
COL4A4	1.077654	7.19E-09	1.15E-07
COL8A2	1.279515	1.03E-15	3.70E-14
CPVL	1.315897	2.36E-17	1.07E-15
CRTAM	1.782859	8.14E-27	2.53E-24
CSF1R	1.391087	2.03E-26	5.29E-24
CSF2RA	1.285642	1.26E-16	5.11E-15
CST7	1.973947	1.91E-23	2.22E-21
CSTA	1.501874	2.77E-17	1.24E-15
CTA-246H3.1	1.368205	1.91E-14	5.86E-13
CTSC	1.09485	1.04E-21	8.73E-20
CTSH	1.746276	3.47E-26	8.37E-24
CTSS	1.349669	1.98E-20	1.35E-18
CTSW	1.021756	5.24E-22	4.64E-20
CTSZ	1.098186	7.71E-10	1.42E-08
CUX2	-1.04847	0.000344	0.002028
CX3CR1	1.272192	3.74E-15	1.25E-13
CXCL1	1.086185	8.99E-07	1.08E-05
CXCL10	2.303445	1.49E-21	1.21E-19
CXCL11	1.846733	3.95E-18	1.95E-16
CXCL12	1.044494	2.18E-13	5.98E-12
CXCL13	1.996558	7.90E-14	2.27E-12
CXCL14	1.99886	2.12E-08	3.19E-07
CXCL2	1.068337	2.15E-08	3.22E-07
CXCL9	2.472737	5.90E-19	3.29E-17
CXCR3	1.954172	1.00E-25	2.08E-23
CXCR5	2.09668	7.79E-15	2.53E-13
CXCR6	1.577563	9.25E-20	5.80E-18
CXorf21	1.174116	7.57E-25	1.26E-22

CYBB	1.176739	1.41E-24	2.08E-22
CYP1B1	1.132925	8.82E-08	1.22E-06
CYR61	1.091829	9.12E-06	8.75E-05
CYTIP	2.113889	1.73E-30	4.39E-27
DAPP1	1.369013	4.13E-25	7.46E-23
DCAL1	1.183426	2.45E-12	5.99E-11
DCN	1.855736	1.18E-11	2.65E-10
DEF6	1.297752	1.18E-23	1.44E-21
DENND1C	1.310998	1.34E-25	2.74E-23
DENND2D	1.702031	8.08E-24	1.05E-21
DHRS9	1.13662	1.20E-13	3.36E-12
DHX58	1.050605	2.43E-18	1.23E-16
DLK1	-1.00159	0.002349	0.009732
DNASE1L3	1.818935	7.16E-16	2.62E-14
DNTT	1.009523	0.000951	0.004683
DOCK2	1.038236	3.88E-18	1.92E-16
DOCK8	1.374375	4.85E-27	1.62E-24
DPEP2	1.300631	9.29E-25	1.51E-22
DPT	1.540962	6.69E-11	1.39E-09
ECGF1	1.313084	1.31E-20	9.19E-19
EDG6	1.809665	1.45E-25	2.92E-23
EFEMP1	1.585677	2.98E-09	5.03E-08
EGR2	1.179517	4.83E-10	9.02E-09
EMP3	1.044263	1.85E-20	1.27E-18
EOMES	2.147984	4.37E-22	3.95E-20
EPHA1	1.176655	1.07E-10	2.18E-09
EPSTI1	1.221995	2.35E-19	1.39E-17
ERP27	1.01241	1.43E-16	5.75E-15
ETV7	1.101181	6.83E-15	2.22E-13
EVI2A	1.369941	1.30E-27	5.36E-25
EVI2B	1.245231	9.57E-26	2.02E-23
EYA4	-1.13304	0.001298	0.005997
FAM113B	1.229379	2.94E-19	1.71E-17
FAM129C	1.043773	2.77E-11	5.97E-10
FAM20A	1.130124	6.74E-14	1.96E-12
FAM26F	1.458018	2.12E-23	2.44E-21
FAM78A	1.301423	4.28E-22	3.89E-20
FASLG	1.130747	1.10E-20	7.86E-19
FBLN5	1.065952	1.10E-08	1.73E-07
FCER1A	1.412901	2.40E-10	4.66E-09
FCGR2B	1.046636	1.68E-13	4.63E-12
FCN1	1.28854	1.06E-21	8.86E-20
FCRL2	1.21421	2.55E-11	5.52E-10
FCRL3	1.339564	9.48E-13	2.42E-11

FCRL5	1.375599	1.60E-12	4.02E-11
FCRLA	1.655105	7.42E-12	1.71E-10
FERMT3	1.097192	1.13E-24	1.75E-22
FGD3	1.17419	6.35E-19	3.52E-17
FGL2	1.509086	1.52E-23	1.79E-21
FLI1	1.205304	5.84E-25	9.94E-23
FLJ20035	1.062215	2.39E-14	7.28E-13
FLJ21438	1.667998	2.25E-26	5.69E-24
FLJ22662	1.110349	2.65E-19	1.57E-17
FLJ22814	1.320278	1.62E-13	4.50E-12
FLJ32255	1.112433	1.55E-20	1.07E-18
FLJ45422	1.185453	4.35E-23	4.69E-21
FLT3	1.514563	1.58E-19	9.62E-18
FMO3	1.352839	1.73E-11	3.81E-10
FMOD	1.394445	5.01E-13	1.32E-11
FPR1	1.052399	4.69E-11	9.86E-10
FUCA1	1.118055	2.36E-13	6.42E-12
FXYD5	1.127277	6.31E-20	4.07E-18
FYB	1.691987	3.05E-31	8.99E-28
GAB3	1.046019	7.17E-22	6.16E-20
GADD45B	1.001986	4.56E-11	9.61E-10
GBP1	1.391137	4.45E-19	2.54E-17
GBP2	1.128633	1.64E-22	1.65E-20
GBP4	1.161247	6.19E-20	4.01E-18
GBP5	1.976561	5.72E-28	3.07E-25
GCET2	1.123588	1.24E-12	3.14E-11
GCGR	1.207011	9.27E-05	0.000676
GFI1	1.63965	1.01E-24	1.60E-22
GGT5	1.09561	1.39E-13	3.88E-12
GGTA1	1.137311	6.58E-17	2.80E-15
GHRL	1.126591	5.50E-16	2.05E-14
GIMAP1	1.377577	8.08E-25	1.34E-22
GIMAP2	1.565442	2.59E-25	4.82E-23
GIMAP4	1.367482	1.30E-27	5.36E-25
GIMAP5	1.486502	4.83E-26	1.07E-23
GIMAP6	1.174047	3.06E-23	3.42E-21
GIMAP7	1.491245	1.91E-25	3.71E-23
GIMAP8	1.049254	2.10E-19	1.26E-17
GLI1	1.069128	4.26E-07	5.37E-06
GMFG	1.339155	4.76E-28	2.63E-25
GNLY	1.38904	1.36E-18	7.08E-17
GP1BA	1.07403	4.53E-15	1.49E-13
GPR157	1.023136	2.71E-13	7.32E-12
GPR171	1.864536	4.71E-25	8.24E-23

GPR174	1.327278	2.74E-10	5.28E-09
GPR18	1.981186	1.57E-20	1.08E-18
GPR183	1.678903	4.21E-19	2.41E-17
GPR65	1.478482	1.32E-24	1.99E-22
GRAP2	1.069529	2.86E-12	6.89E-11
GSDMD	1.140331	3.92E-21	2.95E-19
GZMA	2.198702	5.91E-29	5.48E-26
GZMB	1.636387	1.73E-22	1.73E-20
GZMH	1.326537	3.61E-16	1.37E-14
GZMK	2.62668	9.96E-27	2.99E-24
HCLS1	1.589541	5.78E-32	3.04E-28
HCST	1.740928	1.45E-32	1.28E-28
HLA-A	1.063241	1.82E-26	4.88E-24
HLA-B	1.423826	3.87E-27	1.32E-24
HLA-C	1.139267	2.17E-28	1.48E-25
HLA-DMA	1.397075	3.14E-29	3.27E-26
HLA-DMB	1.605283	1.23E-27	5.36E-25
HLA-DPA1	1.628894	1.48E-25	2.95E-23
HLA-DPB1	1.641603	7.53E-28	3.81E-25
HLA-DPB2	1.038337	2.19E-16	8.62E-15
HLA-DQA1	1.086953	6.67E-07	8.20E-06
HLA-DQA2	1.220897	1.57E-11	3.47E-10
HLA-DQB2	1.463511	1.43E-21	1.17E-19
HLA-DRA	1.378541	8.78E-30	1.41E-26
HLA-DRB1	1.696527	6.19E-29	5.48E-26
HLA-DRB3	1.735389	4.97E-24	6.87E-22
HLA-DRB4	1.657667	9.59E-25	1.54E-22
HLA-DRB5	1.721749	1.25E-28	9.59E-26
HLA-E	1.356634	1.52E-26	4.35E-24
HLA-F	1.549949	6.86E-32	3.04E-28
HLA-H	1.419672	1.42E-22	1.44E-20
HSD11B1	1.057457	8.40E-10	1.54E-08
HSH2D	1.257334	4.83E-18	2.36E-16
IBRDC2	1.007387	2.54E-16	9.88E-15
ICAM1	1.287022	5.82E-24	7.92E-22
ICAM3	1.189445	9.87E-24	1.23E-21
ICOS	1.49181	4.13E-23	4.48E-21
IFI16	1.108759	1.13E-18	5.97E-17
IFI30	1.215631	4.34E-15	1.44E-13
IFI44L	1.453206	6.18E-14	1.80E-12
IFI6	1.481333	5.36E-16	2.00E-14
IFIH1	1.27492	9.76E-24	1.23E-21
IFIT1	1.047918	9.08E-10	1.65E-08
IFITM1	1.603756	2.37E-25	4.56E-23

IFITM3	1.110856	3.35E-18	1.69E-16
IFITM4P	1.138276	3.88E-18	1.92E-16
IFNG	1.367749	1.28E-11	2.87E-10
IGF1	1.147098	2.40E-12	5.88E-11
IGH@	2.717595	4.26E-12	1.00E-10
IGHA1	1.810251	3.74E-12	8.87E-11
IGHD	1.358014	2.43E-10	4.71E-09
IGHM	1.959653	2.06E-17	9.43E-16
IGHV1-69	1.410895	6.56E-15	2.14E-13
IGJ	1.828664	5.27E-16	1.97E-14
IGKC	2.263219	3.24E-14	9.72E-13
IGKV1-5	1.384642	6.33E-10	1.17E-08
IGKV1D-8	1.61291	2.62E-11	5.66E-10
IGL@	1.43635	2.82E-12	6.80E-11
IGSF6	1.01301	1.51E-14	4.69E-13
IKBKE	1.302523	3.43E-16	1.31E-14
IKZF1	1.589073	1.43E-23	1.70E-21
IKZF3	1.20485	2.05E-09	3.53E-08
IL18	1.510967	1.56E-15	5.44E-14
IL18R1	1.343899	7.06E-17	2.99E-15
IL18RAP	1.507265	1.78E-26	4.85E-24
IL1B	1.363297	1.17E-13	3.29E-12
IL21R	1.527029	4.09E-23	4.47E-21
IL28RA	1.643022	2.24E-11	4.88E-10
IL2RB	1.791732	2.66E-26	6.62E-24
IL2RG	1.302361	1.65E-18	8.50E-17
IL32	1.245364	1.38E-19	8.51E-18
IL33	1.289884	1.69E-15	5.87E-14
IL4I1	1.643651	1.78E-15	6.16E-14
IL4R	1.170918	1.13E-20	8.03E-19
IL6	1.017386	8.87E-08	1.22E-06
IL6R	1.506319	4.34E-28	2.48E-25
IL7R	1.964184	7.59E-21	5.53E-19
INCA	1.110709	3.02E-23	3.41E-21
INDO	1.318123	3.32E-16	1.27E-14
INDOL1	2.356304	3.61E-17	1.59E-15
IRF1	1.046685	1.28E-19	7.94E-18
IRF4	1.415404	8.41E-20	5.33E-18
IRF5	1.001848	3.25E-17	1.43E-15
IRF8	1.584726	1.46E-20	1.02E-18
ISG20	1.677509	8.25E-25	1.35E-22
ITGAL	1.214204	4.62E-26	1.05E-23
ITGAX	1.273607	1.79E-21	1.43E-19
ITGB2	1.163203	1.69E-23	1.98E-21

ITGB7	1.530903	3.17E-26	7.80E-24
ITK	2.055831	2.22E-20	1.51E-18
ITM2A	1.591712	1.02E-20	7.33E-19
ITPR3	1.220037	7.71E-17	3.23E-15
KIAA0125	1.751699	3.38E-10	6.44E-09
KLHL6	1.258195	2.98E-24	4.22E-22
KLRB1	1.650551	2.52E-22	2.44E-20
KLRC2	1.313687	2.05E-09	3.53E-08
KLRC3	1.348589	1.19E-10	2.42E-09
KLRD1	1.298805	2.65E-13	7.17E-12
KLRF1	1.361217	9.69E-14	2.75E-12
KLRK1	1.44407	2.62E-17	1.18E-15
KRT222P	1.218356	1.79E-07	2.37E-06
KYNU	1.215244	9.05E-19	4.91E-17
LAD1	1.78296	3.52E-13	9.40E-12
LAIR1	1.081508	1.61E-24	2.35E-22
LAIR2	1.045412	1.15E-07	1.57E-06
LAMC2	1.169387	6.11E-13	1.60E-11
LAMP3	1.774044	4.88E-23	5.20E-21
LAPTM5	1.188317	4.84E-22	4.32E-20
LAX1	1.226968	1.20E-18	6.26E-17
LCK	2.07057	1.23E-26	3.63E-24
LCP1	1.580502	9.87E-24	1.23E-21
LCP2	1.550942	3.59E-34	6.34E-30
LEF1	1.247734	2.63E-19	1.56E-17
LGALS2	2.520429	5.04E-22	4.48E-20
LGALS9	1.02924	4.71E-25	8.24E-23
LILRB1	1.154884	9.14E-19	4.94E-17
LILRB2	1.296595	6.16E-22	5.40E-20
LOC120376	1.485956	8.67E-10	1.59E-08
LOC123862	1.149927	1.58E-18	8.17E-17
LOC158830	1.766132	3.84E-21	2.92E-19
LOC202134	1.080625	0.001326	0.006112
LOC283663	1.033175	4.08E-12	9.60E-11
LOC339192	1.003979	3.60E-06	3.78E-05
LOC402505	-1.00081	0.013699	0.040081
LOC439949	1.645111	1.81E-16	7.19E-15
LOC641518	1.297253	3.63E-12	8.64E-11
LOC642652	1.031667	8.68E-14	2.48E-12
LOC727811	1.100063	5.14E-11	1.07E-09
LST1	1.372119	3.71E-26	8.63E-24
LTB	2.179282	2.78E-19	1.64E-17
LUM	1.989963	6.59E-13	1.72E-11
LY75	1.723718	3.83E-19	2.20E-17

LY86	1.010119	1.31E-22	1.34E-20
LY96	1.166339	1.24E-24	1.89E-22
LYZ	1.704789	5.22E-16	1.96E-14
MAG1	1.178341	1.13E-18	5.97E-17
MAL	1.758758	6.25E-16	2.31E-14
MAN1C1	1.145954	1.03E-13	2.93E-12
MAP3K8	1.082652	1.64E-15	5.69E-14
MARCO	1.270961	4.37E-06	4.48E-05
MBOAT1	1.037605	1.18E-15	4.21E-14
MCOLN2	1.321575	3.58E-17	1.58E-15
MDFIC	1.009046	4.09E-17	1.78E-15
MEOX1	1.017188	1.19E-09	2.11E-08
MEOX2	1.432393	4.23E-10	7.96E-09
MFAP5	1.075878	5.50E-05	0.000429
MFNG	1.299396	1.46E-26	4.22E-24
MGC29506	1.994876	8.79E-18	4.19E-16
MLKL	1.054339	7.64E-20	4.86E-18
MMP9	1.664156	1.63E-10	3.24E-09
MNDA	1.398165	8.60E-24	1.10E-21
MOXD1	1.588409	1.43E-11	3.18E-10
MPEG1	1.141648	1.05E-25	2.15E-23
MS4A1	1.750647	1.38E-12	3.47E-11
MS4A4A	1.103668	4.91E-15	1.62E-13
MX1	1.39374	1.02E-15	3.68E-14
MX2	1.092455	2.53E-15	8.58E-14
MYO1G	1.006842	6.96E-22	6.01E-20
NAPSB	1.517523	2.42E-22	2.37E-20
NCF1	1.258172	1.96E-27	7.71E-25
NCF2	1.240455	1.12E-20	7.99E-19
NCF4	1.179045	1.17E-17	5.53E-16
NCKAP1L	1.36566	9.25E-23	9.52E-21
NCR1	1.543904	3.90E-15	1.29E-13
NCR3	1.053509	1.00E-16	4.15E-15
NEUROD1	-1.07291	0.000596	0.003189
NFATC1	1.080051	6.43E-20	4.14E-18
NGFR	1.087131	4.80E-08	6.89E-07
NKG7	1.696933	2.08E-26	5.34E-24
NLRC5	1.033111	9.96E-27	2.99E-24
NLRP7	1.038783	4.63E-07	5.79E-06
NNMT	1.26987	2.94E-14	8.92E-13
NOD2	1.285945	4.09E-26	9.41E-24
NPC2	1.076248	4.78E-23	5.12E-21
NPL	1.028372	6.91E-19	3.82E-17
OAS1	1.014235	5.80E-12	1.35E-10

OASL	1.195793	1.55E-17	7.25E-16
OCA2	1.194167	1.93E-06	2.16E-05
OGN	1.137078	0.000248	0.001544
OMD	1.509522	8.00E-08	1.11E-06
P2RY10	1.128729	2.76E-09	4.67E-08
P2RY13	1.054579	1.33E-16	5.37E-15
P2RY14	1.067701	5.01E-13	1.32E-11
P2RY8	1.25374	5.31E-20	3.46E-18
PAMCI	1.262816	7.21E-08	1.01E-06
PARP9	1.131685	6.58E-17	2.80E-15
PDCD1	1.063513	1.10E-18	5.85E-17
PDPN	1.376713	1.60E-13	4.44E-12
PIK3CG	1.208643	6.69E-16	2.47E-14
PIP5K1B	1.114697	3.43E-07	4.36E-06
PLA1A	1.096595	2.47E-16	9.65E-15
PLA2G7	1.244584	1.66E-14	5.13E-13
PLAC8	2.0323	6.01E-24	8.06E-22
PLCG2	1.321673	1.13E-29	1.66E-26
PLEK	1.622363	7.64E-29	6.44E-26
PLSCR1	1.115126	9.51E-21	6.87E-19
POU2AF1	2.714865	2.32E-18	1.19E-16
PRKCQ	1.62271	2.67E-18	1.35E-16
PRSS12	-1.04695	5.87E-05	0.000453
PSCD4	1.282857	1.54E-28	1.09E-25
PSD4	1.561508	4.92E-21	3.63E-19
PSMB8	1.264052	2.82E-22	2.67E-20
PSMB9	1.323189	1.06E-27	4.87E-25
PSTPIP1	1.326029	5.75E-23	6.03E-21
PTAFR	1.021828	5.90E-18	2.85E-16
PTGDS	2.411042	9.60E-22	8.17E-20
PTPLAD2	1.258687	5.11E-20	3.35E-18
PTPN22	1.418436	1.20E-23	1.46E-21
PTPN6	1.29817	7.69E-27	2.43E-24
PTPN7	1.15209	6.01E-24	8.06E-22
PTPRC	1.54102	1.38E-24	2.06E-22
PTPRCAP	1.101689	3.67E-18	1.84E-16
PYHIN1	1.472248	2.89E-19	1.69E-17
RAB37	1.696668	3.69E-13	9.82E-12
RAC2	1.358794	9.43E-29	7.58E-26
RARRES2	1.274561	1.33E-14	4.17E-13
RARRES3	1.443653	2.21E-29	2.60E-26
RASSF5	1.301538	1.49E-23	1.77E-21
RBP5	1.292011	2.92E-16	1.13E-14
RCAN3	1.094939	1.86E-06	2.10E-05

RCSD1		1.136105	1.04E-20	7.44E-19
RELB		1.038159	3.78E-18	1.88E-16
RGS1		1.298673	4.33E-12	1.02E-10
RGS18		1.171555	1.97E-23	2.28E-21
RHOH		2.102465	1.66E-26	4.60E-24
RIPK3		1.167921	2.08E-21	1.65E-19
RNASE6		1.095995	3.69E-21	2.83E-19
RSAD2		1.07144	1.46E-10	2.93E-09
RSPO3		1.341072	2.61E-16	1.01E-14
RTP4		1.122942	1.17E-18	6.16E-17
RUNX3		1.46482	2.38E-28	1.56E-25
S100A10		1.156034	1.42E-15	4.99E-14
S100A4		1.102403	4.47E-17	1.94E-15
S100A8		1.235875	1.35E-06	1.57E-05
S100A9		1.044996	3.91E-07	4.94E-06
SAMD3		1.657738	1.15E-21	9.47E-20
SAMD9L		1.313953	4.37E-18	2.15E-16
SAMSN1		1.299557	3.34E-20	2.21E-18
SELE		1.356875	1.97E-09	3.42E-08
SELL		2.345855	1.72E-16	6.84E-15
SELP		1.552617	9.24E-14	2.63E-12
SELPLG		1.016081	2.65E-20	1.78E-18
SEPP1		1.160718	4.83E-14	1.42E-12
	1-Sep	1.805744	5.08E-19	2.87E-17
SERPINB9		1.059573	6.82E-17	2.89E-15
SERPINF1		1.005124	2.36E-08	3.53E-07
SFRP4		1.810639	5.30E-08	7.55E-07
SH2D1A		2.370795	1.05E-24	1.64E-22
SIGLECP3		1.528058	4.22E-16	1.59E-14
SIRPG		1.472461	1.06E-23	1.31E-21
SIT1		1.656857	1.41E-19	8.64E-18
SKAP1		1.786116	4.09E-23	4.47E-21
SLA2		1.124636	1.19E-15	4.23E-14
SLAMF1		1.554853	5.52E-24	7.57E-22
SLAMF7		1.716229	1.54E-21	1.24E-19
SLAMF8		1.473148	3.80E-23	4.20E-21
SLC15A3		1.235855	9.70E-22	8.21E-20
SLC1A3		1.12774	2.89E-15	9.75E-14
SLC22A3		1.459134	1.15E-15	4.11E-14
SLC7A7		1.148455	4.50E-21	3.36E-19
SLCO2A1		1.070661	3.80E-13	1.01E-11
SLCO2B1		1.247058	5.86E-22	5.16E-20
SMOC2		1.107315	2.23E-16	8.75E-15
SNAI3		1.035462	4.54E-19	2.58E-17

SOD3	1.060525	3.08E-12	7.41E-11
SP110	1.446481	1.30E-24	1.96E-22
SPINK2	1.523936	1.28E-11	2.87E-10
SQRDL	1.289844	5.02E-25	8.71E-23
SRGN	1.359576	3.66E-21	2.81E-19
STAB2	1.210101	4.03E-08	5.82E-07
STAP1	2.153085	2.20E-13	6.02E-12
STAT6	1.105055	2.34E-13	6.38E-12
STEAP4	1.126736	2.81E-08	4.14E-07
STK17B	1.00141	8.40E-19	4.60E-17
STYK1	1.015029	7.64E-08	1.07E-06
SUSD3	1.701841	3.51E-26	8.37E-24
SYK	1.24033	3.74E-28	2.28E-25
SYTL1	1.090011	1.84E-16	7.30E-15
TAGAP	1.610038	3.87E-27	1.32E-24
TAP1	1.584614	2.67E-30	5.25E-27
TARP	2.077348	5.32E-21	3.91E-19
TBC1D10C	2.208285	9.80E-25	1.56E-22
TBX21	1.441162	2.31E-23	2.63E-21
TCIRG1	1.018795	1.48E-18	7.68E-17
TCL1A	2.210309	1.21E-15	4.28E-14
TFEC	1.156907	1.84E-17	8.55E-16
TFF3	1.351849	8.94E-11	1.83E-09
TGFBR2	1.207443	1.38E-19	8.51E-18
THBD	1.111732	7.56E-13	1.96E-11
TIMD4	2.180438	1.52E-14	4.72E-13
TLR2	1.325345	1.15E-21	9.47E-20
TLR8	1.837973	5.22E-19	2.94E-17
TMC6	1.254031	1.91E-24	2.76E-22
TMC8	1.102093	2.81E-20	1.87E-18
TMEM154	1.442676	1.76E-13	4.84E-12
TMEM156	1.331465	2.16E-12	5.35E-11
TMEM16J	1.327148	1.36E-16	5.50E-15
TMEM173	1.033422	1.98E-19	1.19E-17
TMEM71	1.429673	9.23E-16	3.33E-14
TNFAIP2	1.289318	2.03E-24	2.92E-22
TNFAIP3	1.333016	1.13E-16	4.62E-15
TNFAIP8L2	1.244208	3.02E-27	1.11E-24
TNFRSF14	1.276056	4.18E-25	7.46E-23
TNFRSF17	1.635038	3.87E-14	1.15E-12
TNFRSF9	1.357762	1.89E-12	4.69E-11
TNFSF10	1.124455	6.58E-18	3.15E-16
TNFSF11	1.117776	9.20E-10	1.67E-08
TNFSF12-TNFSF13	1.047484	1.61E-16	6.40E-15

TNFSF13B	1.185415	4.05E-28	2.39E-25
TNFSF14	1.421089	8.71E-21	6.32E-19
TNIP3	1.010229	4.95E-08	7.08E-07
TP63	1.291314	1.68E-12	4.18E-11
TRAC	2.333174	3.55E-26	8.37E-24
TRAF3IP3	1.408532	2.97E-22	2.80E-20
TRAT1	1.985472	5.79E-19	3.24E-17
TRD@	1.749991	3.12E-21	2.41E-19
TRIM22	1.457159	2.03E-26	5.29E-24
TRIM38	1.00834	5.09E-16	1.91E-14
TTC16	1.079205	1.97E-10	3.87E-09
TXK	1.359138	7.18E-14	2.08E-12
TYROBP	1.178945	2.80E-22	2.66E-20
UBASH3A	2.140215	1.08E-21	9.00E-20
UBD	2.720495	4.12E-21	3.09E-19
VAMP5	1.009504	1.18E-20	8.29E-19
VAMP8	1.326134	3.46E-27	1.25E-24
VAV1	1.469542	2.35E-27	8.86E-25
VCAM1	1.029558	5.99E-15	1.96E-13
VDR	1.512653	2.79E-17	1.24E-15
VENTX	1.01044	3.74E-09	6.23E-08
VNN2	1.067324	1.69E-19	1.02E-17
VSIG9	1.576497	1.17E-18	6.16E-17
WDFY4	1.742713	9.90E-28	4.74E-25
XAF1	1.023948	1.05E-13	2.97E-12
ZAP70	1.871472	3.65E-23	4.06E-21
ZBTB32	1.16471	2.08E-08	3.14E-07
ZC3H12A	1.108607	1.55E-19	9.47E-18
ZC3H12D	1.494732	2.43E-15	8.26E-14
ZFP36	1.270273	8.13E-09	1.30E-07
ZNF683	1.646569	8.72E-19	4.75E-17

DEGs: differentially expressed genes; FDR: false discovery rate.

Table S2. 503 DEGs found between stromal score groups (FDR<0.05).

gene	logFC	pvalue	FDR
A2M	1.099881	1.27E-19	2.32E-17
ABCA6	1.262225	2.60E-12	7.19E-11
ABCA8	1.29917	2.35E-08	3.46E-07
ABCA9	1.238299	4.46E-12	1.17E-10
ABCC3	1.014982	4.29E-10	8.24E-09
ACTA2	1.105258	3.76E-12	1.01E-10
ADAM28	1.078833	2.51E-12	6.94E-11
ADAMTS5	1.059452	1.41E-12	4.06E-11
ADAMTSL3	1.016194	7.18E-14	2.62E-12
ADCY4	1.098842	1.16E-17	1.04E-15
ADCY8	-1.0702	0.005205	0.018306
ADH1A	1.296123	1.43E-08	2.17E-07
ADH1C	1.05174	7.13E-08	9.80E-07
AHR	1.180212	9.62E-18	8.89E-16
AIF1	1.254334	8.01E-22	3.54E-19
AIM1	1.017478	1.98E-12	5.54E-11
ALDH1A1	1.319642	8.54E-14	3.05E-12
ALOX5AP	1.079474	1.95E-10	3.92E-09
AMICA1	1.315874	5.16E-12	1.34E-10
ANGPTL1	1.028748	4.53E-14	1.74E-12
ANGPTL4	1.243406	4.61E-08	6.54E-07
ANXA1	1.403413	8.25E-25	1.56E-21
AOAH	1.006952	9.44E-11	2.03E-09
AOC3	1.116703	5.74E-22	2.60E-19
APBB1IP	1.046401	6.74E-14	2.48E-12
APOD	1.671286	3.31E-10	6.45E-09
APOL3	1.151172	3.37E-17	2.71E-15
AQP1	1.024716	7.06E-17	5.34E-15
ARHGAP15	1.160196	6.33E-14	2.35E-12
ARHGAP9	1.03913	3.51E-10	6.81E-09
ARHGDIB	1.148838	1.55E-21	5.52E-19
ARID5A	1.011662	1.18E-17	1.06E-15
ARRDC2	1.002453	1.58E-15	8.42E-14
ASB9	1.006854	1.24E-14	5.39E-13
ASPN	1.539703	1.10E-14	4.82E-13
ATP1A2	1.189566	2.01E-06	2.08E-05
BACE2	1.099721	1.39E-18	1.62E-16
BATF	1.0262	5.74E-11	1.27E-09
BCL3	1.063945	2.85E-14	1.16E-12
BIN2	1.054016	1.19E-11	2.90E-10
BIRC3	1.069004	4.60E-06	4.44E-05
BLNK	1.16237	1.57E-09	2.78E-08

BST2	1.232883	3.74E-17	2.99E-15
C10orf116	1.337963	3.80E-13	1.21E-11
C10orf56	1.420385	6.65E-23	5.57E-20
C14orf56	1.085271	1.52E-08	2.29E-07
C16orf30	1.118447	4.41E-22	2.16E-19
C1QA	1.25961	1.35E-19	2.41E-17
C1QB	1.206567	1.24E-18	1.48E-16
C1QC	1.045311	7.90E-14	2.85E-12
C1R	1.659841	3.66E-21	1.05E-18
C1S	1.669958	3.08E-17	2.53E-15
C1orf54	1.16542	3.22E-23	3.20E-20
C21orf96	1.113942	4.50E-11	1.01E-09
C3	1.408471	3.10E-11	7.15E-10
C4orf18	1.263191	1.08E-17	9.85E-16
C6orf192	1.044756	9.14E-19	1.16E-16
C6orf97	1.100431	1.92E-10	3.88E-09
C7	1.204373	3.30E-08	4.76E-07
C8orf4	1.123099	5.89E-15	2.74E-13
CABP7	1.118184	0.004529	0.016415
CARD6	1.193644	1.22E-28	1.99E-24
CASP1	1.15147	5.08E-19	6.97E-17
CASP4	1.207147	4.47E-24	5.78E-21
CASP5	1.140836	1.22E-24	2.00E-21
CAV1	1.000218	7.54E-15	3.39E-13
CCDC48	1.052275	2.35E-14	9.69E-13
CCDC80	1.516162	1.29E-18	1.53E-16
CCL11	1.861074	4.57E-14	1.75E-12
CCL13	1.197044	5.30E-10	1.01E-08
CCL14	1.468544	6.06E-12	1.55E-10
CCL18	1.255532	8.48E-07	9.48E-06
CCL19	1.334038	0.000324	0.001864
CCL2	1.395315	7.22E-13	2.19E-11
CCL26	1.048819	8.51E-10	1.58E-08
CCL5	1.114812	8.54E-09	1.34E-07
CCL8	1.304656	2.99E-10	5.86E-09
CCR2	1.124849	6.51E-09	1.04E-07
CCR7	1.349527	8.90E-06	8.04E-05
CD109	1.134577	1.51E-22	1.02E-19
CD163	1.529312	1.10E-19	2.07E-17
CD1C	1.030857	1.26E-06	1.35E-05
CD2	1.314424	1.72E-09	3.02E-08
CD28	1.364685	3.33E-11	7.63E-10
CD37	1.090223	7.37E-09	1.17E-07
CD38	1.066661	7.51E-10	1.40E-08

CD3D	1.237299	6.19E-07	7.09E-06
CD3G	1.134347	2.03E-07	2.57E-06
CD48	1.048833	1.03E-07	1.37E-06
CD52	1.182197	1.74E-07	2.24E-06
CD53	1.094377	1.48E-10	3.07E-09
CD69	1.347587	1.98E-07	2.52E-06
CD74	1.214551	2.62E-11	6.12E-10
CD86	1.077354	8.09E-08	1.10E-06
CD93	1.007282	7.07E-18	6.75E-16
CDH19	1.059073	0.000933	0.004528
CEBPD	1.201229	9.00E-16	5.20E-14
CFB	1.126277	1.11E-14	4.85E-13
CFD	1.200414	3.43E-16	2.21E-14
CFH	1.304551	5.54E-21	1.50E-18
CFHR3	1.48129	1.82E-21	6.12E-19
CFI	1.095989	3.80E-14	1.50E-12
CH25H	1.182991	2.56E-10	5.10E-09
CHI3L2	1.151817	5.98E-05	0.000443
CILP	1.183872	8.46E-11	1.83E-09
CLEC14A	1.044494	3.48E-18	3.58E-16
CLEC2B	1.175648	2.86E-24	4.32E-21
CLEC3B	1.224018	2.25E-15	1.17E-13
CLIC5	1.036432	4.68E-14	1.79E-12
CLIC6	1.05681	5.28E-06	5.02E-05
CNN2	1.231452	8.25E-20	1.63E-17
COL10A1	1.343221	8.90E-12	2.22E-10
COL12A1	1.4947	1.78E-22	1.08E-19
COL14A1	1.245308	7.37E-23	5.57E-20
COL15A1	1.098267	2.36E-13	7.79E-12
COL16A1	1.211848	1.43E-16	1.03E-14
COL1A1	1.715198	3.45E-19	5.17E-17
COL1A2	1.567646	6.93E-23	5.57E-20
COL3A1	1.661618	2.58E-22	1.46E-19
COL4A2	1.013362	6.30E-16	3.81E-14
COL5A1	1.479256	2.89E-19	4.55E-17
COL5A2	1.277807	1.07E-22	7.75E-20
COL5A3	1.024949	4.82E-13	1.50E-11
COL6A2	1.176054	1.24E-20	3.00E-18
COL6A3	1.244655	5.29E-18	5.30E-16
COL8A1	1.624025	3.47E-13	1.11E-11
COL8A2	1.214413	2.97E-14	1.21E-12
COMP	1.361478	3.00E-08	4.35E-07
COPZ2	1.272358	6.30E-21	1.68E-18
CPVL	1.175384	5.29E-13	1.64E-11

CPXM2	1.29738	2.76E-14	1.13E-12
CPZ	1.323313	1.95E-12	5.49E-11
CRISPLD2	1.008843	9.96E-15	4.40E-13
CRTAM	1.097845	2.92E-09	4.96E-08
CSDA	1.075663	8.74E-20	1.70E-17
CSF1R	1.298127	2.83E-21	8.68E-19
CTGF	1.645996	3.37E-16	2.18E-14
CTSH	1.04219	3.47E-09	5.81E-08
CTSK	1.106551	9.99E-21	2.48E-18
CTSS	1.025814	1.14E-10	2.41E-09
CX3CR1	1.157374	3.60E-12	9.67E-11
CXCL1	1.02205	1.68E-06	1.77E-05
CXCL10	1.376372	2.25E-08	3.33E-07
CXCL12	1.122287	2.11E-15	1.10E-13
CXCL14	1.618217	4.64E-06	4.47E-05
CXCL2	1.048621	8.00E-08	1.09E-06
CXCL9	1.288824	2.65E-06	2.70E-05
CXCR3	1.12032	6.05E-09	9.73E-08
CXCR6	1.01089	9.42E-09	1.47E-07
CYBRD1	1.267377	2.61E-28	1.99E-24
CYP1B1	1.286515	7.32E-10	1.37E-08
CYR61	1.455422	1.38E-09	2.45E-08
CYTIP	1.190828	3.81E-09	6.33E-08
DAB2	1.135339	7.19E-26	2.17E-22
DCN	2.408326	1.64E-20	3.92E-18
DES	1.056107	3.60E-07	4.36E-06
DPT	1.493576	2.95E-10	5.79E-09
ECM2	1.00254	2.06E-19	3.52E-17
EDG6	1.085227	3.40E-09	5.71E-08
EFEMP1	1.985931	5.54E-16	3.41E-14
EGR1	1.026585	1.19E-09	2.15E-08
EGR2	1.525447	3.15E-16	2.05E-14
EMCN	1.164438	2.66E-21	8.32E-19
EMP1	1.457331	7.95E-19	1.03E-16
EMP3	1.059463	6.07E-20	1.26E-17
EOMES	1.044489	7.12E-06	6.59E-05
EPSTI1	1.00274	5.66E-13	1.75E-11
F13A1	1.487574	2.91E-13	9.47E-12
FAM129A	1.050484	1.23E-15	6.80E-14
FAM20A	1.264907	4.31E-17	3.37E-15
FAP	1.656021	5.41E-20	1.15E-17
FBLN2	1.383158	2.87E-18	3.06E-16
FBLN5	1.367502	4.83E-14	1.84E-12
FCER1A	1.147602	6.29E-07	7.20E-06

FCGR1B	1.396075	3.98E-13	1.26E-11
FCGR3A	1.337889	3.94E-11	8.93E-10
FER1L3	1.052454	2.41E-21	7.66E-19
FGL2	1.173819	2.25E-13	7.49E-12
FHL2	1.160259	6.04E-15	2.80E-13
FLI1	1.11331	2.18E-20	5.00E-18
FLJ20035	1.013198	1.53E-12	4.37E-11
FLJ20920	1.127513	1.68E-18	1.88E-16
FLJ21438	1.184239	6.59E-13	2.01E-11
FLJ21963	1.025797	6.25E-16	3.80E-14
FLJ21986	1.252468	8.74E-15	3.88E-13
FLJ22662	1.197164	8.26E-22	3.56E-19
FLJ43692	1.001863	1.09E-06	1.19E-05
FMO2	1.08227	5.98E-11	1.32E-09
FMOD	1.581998	2.21E-16	1.51E-14
FNDC1	1.769678	2.35E-16	1.60E-14
FOS	1.252646	4.29E-07	5.08E-06
FOSB	1.45426	3.58E-06	3.56E-05
FPR1	1.1899	2.36E-13	7.79E-12
FSTL1	1.143776	5.29E-24	6.39E-21
FYB	1.199709	3.63E-14	1.44E-12
GADD45B	1.093789	5.97E-13	1.84E-11
GBP1	1.268945	4.75E-16	2.99E-14
GBP2	1.096498	4.37E-21	1.22E-18
GBP3	1.094458	1.87E-15	9.84E-14
GBP5	1.20047	2.09E-11	4.93E-10
GGT5	1.439203	3.36E-23	3.20E-20
GGTA1	1.210757	3.06E-19	4.70E-17
GGTLA1	1.126429	3.29E-22	1.75E-19
GIMAP1	1.150049	9.19E-17	6.83E-15
GIMAP2	1.114131	5.35E-12	1.38E-10
GIMAP4	1.207026	4.04E-21	1.14E-18
GIMAP5	1.137138	4.60E-15	2.19E-13
GIMAP6	1.063749	4.29E-19	6.07E-17
GIMAP7	1.116769	7.07E-14	2.58E-12
GIMAP8	1.044286	3.06E-19	4.70E-17
GLI1	1.54358	5.08E-15	2.39E-13
GLI3	1.075037	3.51E-21	1.03E-18
GLT8D2	1.001525	1.29E-14	5.58E-13
GLT8D4	1.268164	4.34E-15	2.09E-13
GMFG	1.165272	8.71E-21	2.22E-18
GNG11	1.088247	4.21E-20	9.20E-18
GPIHBP1	1.099659	1.10E-11	2.70E-10
GPM6B	1.035759	5.44E-07	6.30E-06

GPR171	1.016926	2.22E-07	2.79E-06
GPR183	1.021384	5.87E-07	6.74E-06
GPR34	1.024796	7.77E-14	2.81E-12
GPR65	1.169032	2.20E-14	9.12E-13
GRAMD1C	1.001574	4.02E-07	4.81E-06
GZMA	1.299637	5.52E-10	1.05E-08
GZMB	1.050373	4.53E-08	6.43E-07
GZMK	1.381581	3.30E-07	4.03E-06
HCLS1	1.252663	2.82E-18	3.06E-16
HCST	1.021028	2.53E-11	5.93E-10
HES1	1.041218	1.06E-13	3.77E-12
HHEX	1.023394	4.83E-14	1.84E-12
HLA-B	1.075175	3.36E-15	1.67E-13
HLA-DMA	1.102925	4.79E-17	3.73E-15
HLA-DMB	1.213603	3.62E-15	1.77E-13
HLA-DPA1	1.213343	1.68E-13	5.72E-12
HLA-DPB1	1.200405	3.93E-14	1.54E-12
HLA-DRA	1.062591	8.72E-17	6.50E-15
HLA-DRB1	1.205769	4.46E-14	1.72E-12
HLA-DRB3	1.21956	3.42E-11	7.82E-10
HLA-DRB4	1.233802	7.44E-13	2.25E-11
HLA-DRB5	1.225057	4.39E-14	1.70E-12
HLA-E	1.097628	2.16E-16	1.47E-14
HLA-F	1.104512	1.04E-15	5.87E-14
HLA-H	1.07248	4.69E-12	1.22E-10
HMOX1	1.280473	1.25E-10	2.62E-09
HSD11B1	1.10768	7.21E-12	1.83E-10
HTRA1	1.160145	6.49E-20	1.34E-17
HTRA3	1.087939	3.61E-17	2.90E-15
IBRDC2	1.04083	1.29E-17	1.14E-15
IER3	1.018045	7.33E-13	2.22E-11
IFI16	1.129459	3.76E-19	5.54E-17
IFI30	1.060867	3.50E-12	9.43E-11
IFI44	1.032874	1.55E-11	3.71E-10
IFI44L	1.188503	1.12E-09	2.04E-08
IFIH1	1.046954	6.98E-16	4.08E-14
IFITM1	1.367735	1.99E-18	2.21E-16
IFITM2	1.264071	1.87E-23	2.00E-20
IFITM3	1.273765	1.02E-23	1.15E-20
IFITM4P	1.237005	2.73E-20	6.18E-18
IGF1	1.388749	3.17E-18	3.31E-16
IGFBP4	1.114866	1.31E-21	4.75E-19
IGH@	1.374973	0.000509	0.002722
IGJ	1.066367	1.60E-06	1.69E-05

IGKC	1.164181	0.000277	0.001635
IL2RB	1.058779	1.09E-09	1.99E-08
IL32	1.017223	8.93E-13	2.66E-11
IL33	1.048649	5.71E-12	1.47E-10
IL4R	1.096212	6.52E-18	6.32E-16
IL6R	1.139301	2.75E-15	1.39E-13
IL7R	1.06425	5.23E-06	4.98E-05
INDOL1	1.072475	0.000176	0.00112
INHBA	1.39557	7.42E-15	3.34E-13
IRF8	1.018522	1.44E-08	2.18E-07
ISG20	1.240035	1.43E-13	4.98E-12
ISLR	1.324384	1.12E-21	4.31E-19
ITGA11	1.138398	1.45E-16	1.03E-14
ITGBL1	1.119966	2.17E-13	7.24E-12
ITK	1.100968	3.51E-06	3.49E-05
ITM2A	1.300621	2.13E-13	7.16E-12
ITPR3	1.050654	9.48E-13	2.80E-11
KANK1	1.219862	2.08E-21	6.72E-19
KCNE4	1.117637	5.49E-14	2.06E-12
KDELR3	1.167432	6.92E-16	4.06E-14
KIAA1217	1.131936	1.26E-17	1.12E-15
KIAA1913	1.110971	2.16E-17	1.84E-15
KLF4	1.034332	3.11E-09	5.25E-08
LAMA2	1.316042	3.83E-13	1.22E-11
LAMA4	1.022798	2.22E-13	7.39E-12
LAMB2	1.194443	3.16E-22	1.73E-19
LAPTM5	1.012213	4.45E-15	2.12E-13
LATS2	1.133801	1.62E-25	4.19E-22
LCK	1.023812	1.07E-06	1.18E-05
LCP2	1.140124	4.16E-17	3.27E-15
LEF1	1.061885	3.40E-14	1.36E-12
LEPREL1	1.314006	1.10E-20	2.70E-18
LILRB2	1.186599	5.39E-18	5.36E-16
LIX1	-1.00825	0.001106	0.005181
LOC123862	1.265321	5.46E-22	2.54E-19
LOC158830	1.000853	5.87E-07	6.74E-06
LOC391020	1.253905	4.41E-22	2.16E-19
LOC400456	1.057146	2.24E-18	2.45E-16
LOC402505	-1.16428	0.000456	0.00248
LOC493869	1.251602	1.30E-19	2.36E-17
LOC641518	1.250799	1.72E-10	3.52E-09
LOC642652	1.080854	3.46E-14	1.38E-12
LOC651721	1.761304	2.74E-16	1.81E-14
LOX	1.096547	9.70E-13	2.86E-11

LOXL1	1.011765	2.62E-12	7.22E-11
LRRC32	1.098671	9.62E-18	8.89E-16
LST1	1.06034	2.29E-15	1.18E-13
LTBP1	1.165529	5.94E-14	2.21E-12
LTBR	1.001393	1.01E-18	1.27E-16
LUM	2.406492	5.95E-20	1.26E-17
LY75	1.112066	8.57E-08	1.15E-06
LY96	1.058715	8.40E-19	1.07E-16
MAN1C1	1.017624	7.79E-11	1.69E-09
MAP3K8	1.114458	4.00E-15	1.94E-13
MBOAT1	1.121852	1.55E-18	1.76E-16
MDFIC	1.177334	8.61E-25	1.56E-21
MEF2C	1.100697	9.60E-22	3.87E-19
MEOX2	1.449112	4.83E-10	9.26E-09
MFAP4	1.157415	1.48E-07	1.92E-06
MFAP5	1.383779	3.68E-07	4.45E-06
MFNG	1.022296	1.42E-16	1.02E-14
MGC29506	1.212181	2.70E-06	2.75E-05
MGC45438	1.066725	5.44E-10	1.04E-08
MGP	1.572447	8.71E-21	2.22E-18
MGST1	1.048778	1.92E-06	2.00E-05
MLKL	1.024758	4.29E-19	6.07E-17
MN1	1.005995	5.11E-14	1.93E-12
MNDA	1.261698	2.95E-18	3.11E-16
MOXD1	1.204313	8.33E-08	1.13E-06
MRC1L1	1.099177	8.10E-12	2.03E-10
MS4A4A	1.220491	6.46E-18	6.29E-16
MS4A7	1.172597	3.36E-15	1.67E-13
MT1B	1.086894	1.19E-13	4.18E-12
MT1M	1.275926	8.28E-13	2.48E-11
MT1X	1.059193	8.18E-15	3.64E-13
MT2A	1.441752	3.61E-16	2.32E-14
MX1	1.085968	5.27E-09	8.60E-08
MXRA5	1.279502	4.37E-19	6.14E-17
MXRA8	1.126936	2.99E-16	1.95E-14
MYH11	1.002848	6.88E-06	6.38E-05
MYL9	1.191139	1.92E-20	4.47E-18
MYLK	1.037764	6.97E-19	9.30E-17
MYO5C	1.05418	8.15E-14	2.93E-12
NCF2	1.06976	1.02E-15	5.81E-14
NEXN	1.146006	7.79E-20	1.59E-17
NGFR	1.057301	1.06E-06	1.17E-05
NID2	1.229738	4.92E-16	3.06E-14
NKX6-2	-1.14363	0.000409	0.002258

NNMT	1.649894	1.84E-22	1.08E-19
NOTCH2	1.240029	7.41E-25	1.56E-21
NOV	1.19392	4.68E-07	5.49E-06
NR2F2	1.172586	1.37E-22	9.53E-20
NR4A2	1.089946	1.88E-05	0.00016
NUPR1	1.180212	6.12E-18	6.03E-16
NYD-SP21	1.183473	1.53E-15	8.24E-14
OAF	1.056677	3.62E-19	5.38E-17
OGN	2.094644	7.11E-12	1.81E-10
OLFML2B	1.042585	8.17E-18	7.68E-16
OLR1	1.010181	2.28E-05	0.000189
OMD	1.90853	2.29E-13	7.59E-12
OSR2	1.084973	1.98E-12	5.54E-11
P2RY14	1.169578	5.06E-17	3.90E-15
P2RY5	1.133134	1.17E-21	4.43E-19
P2RY8	1.010398	1.12E-12	3.25E-11
PAMCI	1.263651	2.96E-08	4.30E-07
PDGFRL	1.148244	5.61E-09	9.11E-08
PDK4	1.113714	3.04E-12	8.29E-11
PDPN	1.065442	3.67E-08	5.26E-07
PLA2G4A	1.151162	1.47E-18	1.68E-16
PLAC8	1.092989	2.59E-06	2.65E-05
PLAT	1.119499	4.99E-11	1.11E-09
PLAU	1.094995	9.97E-18	9.17E-16
PLEK2	1.00035	1.79E-05	0.000152
PLEKHA4	1.089848	1.27E-15	6.97E-14
PLEKHH2	1.171352	2.79E-16	1.83E-14
PLP1	1.163475	0.000607	0.003156
PLS3	1.119291	1.26E-19	2.32E-17
PLSCR1	1.016028	1.89E-16	1.30E-14
PLSCR4	1.364032	4.20E-22	2.16E-19
PODN	1.591534	1.87E-15	9.84E-14
POSTN	1.413764	8.85E-10	1.64E-08
POU2AF1	1.317445	4.04E-05	0.000314
PPIC	1.490756	3.29E-28	1.99E-24
PRKCQ	1.014818	2.57E-07	3.20E-06
PROCR	1.153073	9.93E-19	1.25E-16
PRRX1	1.181448	9.20E-13	2.73E-11
PRSS23	1.139583	1.62E-21	5.53E-19
PSCD4	1.003968	1.02E-16	7.52E-15
PTGDS	1.027443	7.31E-05	0.000527
PTGS2	1.199129	6.97E-08	9.60E-07
PTPLAD2	1.158475	3.13E-17	2.56E-15
PTRF	1.083643	1.61E-22	1.04E-19

PYGL	1.105301	2.70E-19	4.34E-17
RAB34	1.042004	1.27E-13	4.42E-12
RAB7B	1.109639	2.53E-17	2.12E-15
RARRES2	1.40046	9.43E-17	6.98E-15
RARRES3	1.042961	1.62E-14	6.85E-13
RCSD1	1.069941	8.10E-19	1.04E-16
RFTN2	1.008119	4.43E-14	1.71E-12
RGS1	1.148225	6.68E-09	1.07E-07
RGS18	1.097543	7.11E-19	9.33E-17
RHOH	1.046006	9.18E-07	1.02E-05
RNASE4	1.176991	6.47E-19	8.76E-17
RNASE6	1.040212	4.61E-18	4.70E-16
RSPO3	1.260595	2.75E-13	9.01E-12
RTP4	1.017056	2.33E-15	1.20E-13
S100A10	1.257897	1.05E-18	1.30E-16
S100A11	1.021544	1.94E-19	3.35E-17
S100A16	1.127686	1.62E-21	5.53E-19
S100A4	1.073822	9.55E-16	5.48E-14
S100A8	1.184212	3.76E-06	3.71E-05
S1PR1	1.067625	2.49E-15	1.27E-13
SAMD9L	1.196631	7.18E-15	3.25E-13
SAMSN1	1.080558	5.84E-13	1.80E-11
SASH1	1.092253	6.27E-28	2.84E-24
SDPR	1.037402	8.88E-11	1.91E-09
SELE	1.449737	8.51E-10	1.58E-08
SELL	1.23947	0.00016	0.001033
SELP	1.317255	1.13E-09	2.05E-08
SEPP1	1.332408	6.94E-18	6.70E-16
SERPINF1	1.072059	1.78E-08	2.68E-07
SERTAD1	1.029881	3.31E-17	2.68E-15
SFRP2	1.51263	1.21E-06	1.31E-05
SFRP4	2.608334	6.72E-15	3.06E-13
SH2D1A	1.134216	3.73E-06	3.68E-05
SIGLECP3	1.171485	1.36E-09	2.42E-08
SLAMF8	1.127812	5.25E-13	1.63E-11
SLC15A3	1.112919	4.05E-17	3.19E-15
SLC1A3	1.044465	2.22E-13	7.39E-12
SLCO2A1	1.011132	9.22E-12	2.30E-10
SLCO2B1	1.224427	1.95E-21	6.44E-19
SMOC2	1.041526	1.38E-14	5.90E-13
SOD3	1.27473	2.67E-17	2.22E-15
SP110	1.270817	1.09E-18	1.34E-16
SPARC	1.17785	7.37E-23	5.57E-20
SPHK1	1.047108	2.04E-13	6.87E-12

SPOCD1	1.006031	1.33E-06	1.43E-05
SPON1	1.501812	1.79E-10	3.65E-09
SPON2	1.466616	8.01E-20	1.61E-17
SPRY1	1.098123	5.47E-12	1.41E-10
SQRDL	1.124016	7.44E-19	9.71E-17
SRGN	1.041825	1.68E-12	4.76E-11
SRPX	1.323484	2.44E-19	4.09E-17
SRPX2	1.426411	2.26E-19	3.83E-17
SST	-1.37756	0.000308	0.001781
STAT6	1.128244	1.62E-14	6.85E-13
STEAP4	1.188414	6.09E-10	1.15E-08
SULF1	1.431107	9.08E-16	5.22E-14
SUSD3	1.055144	3.20E-10	6.24E-09
TAGLN	1.164058	7.07E-18	6.75E-16
TAP1	1.048696	1.34E-13	4.65E-12
TARP	1.022484	7.44E-06	6.85E-05
TBC1D10C	1.320897	5.82E-09	9.41E-08
TBC1D8B	1.066583	3.30E-15	1.65E-13
TCL1A	1.062258	0.000425	0.002336
TGFB3	1.20374	1.45E-16	1.03E-14
TGFBI	1.198718	7.67E-18	7.28E-16
TGFBR2	1.14642	1.35E-17	1.19E-15
THBD	1.397819	2.94E-21	8.74E-19
THBS1	1.131443	1.36E-14	5.81E-13
THBS2	1.681161	1.22E-21	4.52E-19
THBS4	1.210538	1.57E-05	0.000135
TIE1	1.099415	6.70E-17	5.10E-15
TIMD4	1.06548	0.000295	0.001726
TIMP1	1.217005	1.90E-15	9.95E-14
TIMP4	1.424901	3.58E-09	5.98E-08
TLR2	1.281096	4.98E-19	6.89E-17
TLR8	1.215526	3.64E-09	6.08E-08
TM4SF1	1.087524	2.78E-12	7.63E-11
TMEM154	1.080326	1.91E-08	2.85E-07
TMEM173	1.183488	5.52E-26	2.00E-22
TMEM47	1.041766	1.05E-14	4.61E-13
TMEM71	1.137998	6.99E-10	1.31E-08
TNFAIP2	1.109407	9.03E-18	8.43E-16
TNFAIP6	1.144842	7.06E-09	1.12E-07
TNFRSF14	1.034409	2.58E-16	1.73E-14
TPM2	1.077982	2.04E-17	1.75E-15
TRAC	1.273906	5.59E-08	7.82E-07
TRIM22	1.178813	2.61E-16	1.74E-14
TRIM38	1.081503	4.29E-19	6.07E-17

TRIM6	1.025626	2.15E-13	7.20E-12
TYROBP	1.136133	1.85E-20	4.35E-18
UACA	1.043948	6.57E-16	3.91E-14
UBASH3A	1.057171	8.28E-06	7.54E-05
VAMP5	1.031098	5.09E-22	2.43E-19
VAMP8	1.133944	1.05E-19	2.00E-17
VDR	1.421932	4.64E-15	2.20E-13
VENTX	1.001552	5.97E-10	1.13E-08
VSIG4	1.518114	2.99E-11	6.94E-10
WDFY4	1.155738	1.66E-11	3.96E-10
WIPI1	1.105547	3.30E-19	5.02E-17
WISP1	1.041988	7.67E-13	2.31E-11
WISP2	1.274085	1.58E-09	2.79E-08
XAF1	1.069819	4.53E-14	1.74E-12
XLKD1	1.320765	6.43E-09	1.03E-07
YAP1	1.006411	1.82E-22	1.08E-19
ZAP70	1.015026	2.39E-07	3.00E-06
ZC3H12A	1.023665	8.20E-16	4.75E-14
ZC3H12D	1.076836	4.37E-08	6.21E-07
ZFP36	1.466362	1.11E-11	2.73E-10
ZNF683	1.090657	2.28E-09	3.91E-08

DEGs: differentially expressed genes; FDR: false discovery rate.

Table S3. The performance of 280 common DEGs in Kaplan–Meier analysis from the E-MTAB-8248 cohort with pvalue tested in log-rank test.

gene	pvalue	prognostic correlation	regulation in TME
RGS1	4.92E-05	positively	up-regulated
CXCR6	0.000115773	positively	up-regulated
CYP1B1	0.000841932	negatively	up-regulated
IL7R	0.001138517	positively	up-regulated
PTGDS	0.002357261	positively	up-regulated
SMOC2	0.00369523	positively	up-regulated
HLA-DMA	0.004146278	positively	up-regulated
CXCL14	0.004282216	negatively	up-regulated
CLIC6	0.004585763	positively	up-regulated
BIRC3	0.005064215	positively	up-regulated
TRAC	0.005455075	positively	up-regulated
GZMK	0.00553001	positively	up-regulated
FGL2	0.007567598	positively	up-regulated
CD69	0.007572367	positively	up-regulated
TLR8	0.008255818	positively	up-regulated
GZMA	0.00900038	positively	up-regulated
IGH@	0.010446264	negatively	up-regulated
GPR183	0.010691506	positively	up-regulated
RHOH	0.01129222	positively	up-regulated
LCK	0.011624719	positively	up-regulated
GPR171	0.013243245	positively	up-regulated
CCL19	0.013557588	positively	up-regulated
ARHGAP15	0.013917259	positively	up-regulated
CD1C	0.013928199	positively	up-regulated
CD52	0.013992397	positively	up-regulated
HLA-DPB1	0.014579249	positively	up-regulated
CD3D	0.014862922	positively	up-regulated
LY75	0.015399805	positively	up-regulated
CCL5	0.015979723	positively	up-regulated
MOXD1	0.016000657	positively	up-regulated
SLAMF8	0.016798116	positively	up-regulated
ITK	0.016816901	positively	up-regulated
TARP	0.017549401	positively	up-regulated
DPT	0.018775297	negatively	up-regulated
CD2	0.020145352	positively	up-regulated
CCL2	0.023365703	positively	up-regulated
UBASH3A	0.023756381	positively	up-regulated
CTSH	0.023956369	positively	up-regulated
HLA-DMB	0.025411505	positively	up-regulated
P2RY14	0.027035438	positively	up-regulated

ADAM28	0.032420669	positively	up-regulated
ARHGAP9	0.033567304	positively	up-regulated
HLA-DRB1	0.033574379	positively	up-regulated
HLA-DRA	0.03425344	positively	up-regulated
EOMES	0.0364909	positively	up-regulated
CD48	0.036556594	positively	up-regulated
HLA-DPA1	0.036978527	positively	up-regulated
IRF8	0.037192141	positively	up-regulated
SH2D1A	0.03740716	positively	up-regulated
BIN2	0.039153107	positively	up-regulated
SIGLECP3	0.039997166	positively	up-regulated
IL32	0.041879616	positively	up-regulated
ABCA9	0.04278403	positively	up-regulated
SEPP1	0.043655597	positively	up-regulated
CRTAM	0.045119766	positively	up-regulated
IL33	0.045894422	positively	up-regulated
GPR65	0.046711575	positively	up-regulated
ZNF683	0.047064133	positively	up-regulated
CCR2	0.047152991	positively	up-regulated
CD74	0.04747393	positively	up-regulated
ABCA6	0.048329644	positively	up-regulated
APBB1IP	0.048479393	positively	up-regulated
FYB	0.048482802	positively	up-regulated
GIMAP2	0.048589211	positively	up-regulated
LCP2	0.053325895	NA	up-regulated
RGS18	0.055494193	NA	up-regulated
AIF1	0.055587472	NA	up-regulated
SLC15A3	0.055895987	NA	up-regulated
CYTIP	0.056826007	NA	up-regulated
PSCD4	0.057453118	NA	up-regulated
LST1	0.057951225	NA	up-regulated
AOAH	0.059845115	NA	up-regulated
CD53	0.060140139	NA	up-regulated
HLA-DRB3	0.060995566	NA	up-regulated
CYR61	0.061507769	NA	up-regulated
LUM	0.064866026	NA	up-regulated
HLA-DRB5	0.066181106	NA	up-regulated
WDFY4	0.067634201	NA	up-regulated
TMEM71	0.070927037	NA	up-regulated
CASP1	0.075839234	NA	up-regulated
CD38	0.077969597	NA	up-regulated
BLNK	0.081688517	NA	up-regulated
CCL18	0.08408572	NA	up-regulated
P2RY8	0.08669877	NA	up-regulated

SERPINF1	0.094377596	NA	up-regulated
SAMD9L	0.099525193	NA	up-regulated
ZAP70	0.100481141	NA	up-regulated
PDPN	0.101130272	NA	up-regulated
SLCO2B1	0.101300961	NA	up-regulated
ITM2A	0.102152881	NA	up-regulated
TLR2	0.102488858	NA	up-regulated
CD37	0.108952179	NA	up-regulated
SELL	0.11041197	NA	up-regulated
CLIC5	0.11044122	NA	up-regulated
LOC641518	0.111288461	NA	up-regulated
TBC1D10C	0.112039192	NA	up-regulated
MAP3K8	0.113584673	NA	up-regulated
C3	0.114988474	NA	up-regulated
CXCL10	0.116833441	NA	up-regulated
AIM1	0.117948256	NA	up-regulated
TYROBP	0.124053156	NA	up-regulated
ALDH1A1	0.127950557	NA	up-regulated
INDOL1	0.131963834	NA	up-regulated
C1S	0.133546875	NA	up-regulated
PTPLAD2	0.134140556	NA	up-regulated
HLA-DRB4	0.13533267	NA	up-regulated
IL2RB	0.135868246	NA	up-regulated
ZFP36	0.136972595	NA	up-regulated
IGKC	0.137119527	NA	up-regulated
HLA-E	0.13850436	NA	up-regulated
ARHGDIB	0.151320131	NA	up-regulated
TGFBR2	0.153994843	NA	up-regulated
S100A4	0.156185158	NA	up-regulated
ALOX5AP	0.156764863	NA	up-regulated
FMOD	0.161988816	NA	up-regulated
SFRP4	0.162187795	NA	up-regulated
NNMT	0.169009607	NA	up-regulated
SOD3	0.169123035	NA	up-regulated
TRIM22	0.173901176	NA	up-regulated
STEAP4	0.175586944	NA	up-regulated
CXCR3	0.181287868	NA	up-regulated
CSF1R	0.181608632	NA	up-regulated
TNFRSF14	0.185392897	NA	up-regulated
SRGN	0.187861748	NA	up-regulated
EDG6	0.19449898	NA	up-regulated
PLAC8	0.200428375	NA	up-regulated
CHI3L2	0.200954167	NA	up-regulated
TIMD4	0.206659129	NA	up-regulated

TCL1A	0.208500855	NA	up-regulated
CCDC80	0.209289959	NA	up-regulated
AHR	0.222548668	NA	up-regulated
LEF1	0.223712662	NA	up-regulated
LOC158830	0.23425455	NA	up-regulated
MDFIC	0.23662091	NA	up-regulated
CLEC2B	0.239611639	NA	up-regulated
IL6R	0.249598359	NA	up-regulated
C1QA	0.250686972	NA	up-regulated
EFEMP1	0.250739055	NA	up-regulated
CD163	0.257005262	NA	up-regulated
RCSD1	0.258254279	NA	up-regulated
MAN1C1	0.260478712	NA	up-regulated
HLA-B	0.266994343	NA	up-regulated
RNASE6	0.267840089	NA	up-regulated
GIMAP8	0.272378303	NA	up-regulated
THBD	0.28002064	NA	up-regulated
C21orf96	0.285055778	NA	up-regulated
MLKL	0.286132013	NA	up-regulated
FAM20A	0.290926044	NA	up-regulated
GIMAP1	0.295033801	NA	up-regulated
IFIH1	0.295964963	NA	up-regulated
CD3G	0.296631675	NA	up-regulated
IFITM3	0.297017698	NA	up-regulated
HCLS1	0.302243963	NA	up-regulated
DCN	0.304616779	NA	up-regulated
GMFG	0.308588889	NA	up-regulated
MBOAT1	0.315863934	NA	up-regulated
LOC123862	0.320907845	NA	up-regulated
ABCC3	0.322982988	NA	up-regulated
CCR7	0.324224934	NA	up-regulated
IFI16	0.328050325	NA	up-regulated
FLI1	0.336119132	NA	up-regulated
CXCL12	0.344220069	NA	up-regulated
HLA-H	0.356823677	NA	up-regulated
FBLN5	0.364748623	NA	up-regulated
APOL3	0.365800709	NA	up-regulated
CXCL1	0.365897264	NA	up-regulated
BCL3	0.380749776	NA	up-regulated
GIMAP5	0.382116336	NA	up-regulated
TRIM38	0.389871742	NA	up-regulated
PRKCQ	0.419327016	NA	up-regulated
RSPO3	0.419832241	NA	up-regulated
SLC1A3	0.421761156	NA	up-regulated

EGR2	0.427433543	NA	up-regulated
IFI44L	0.436143314	NA	up-regulated
VDR	0.438131045	NA	up-regulated
S100A10	0.443693514	NA	up-regulated
TNFAIP2	0.450930614	NA	up-regulated
CCL11	0.455913928	NA	up-regulated
IFI30	0.461649152	NA	up-regulated
C10orf116	0.464054764	NA	up-regulated
TMEM173	0.465109264	NA	up-regulated
FLJ20035	0.465348142	NA	up-regulated
VENTX	0.467044202	NA	up-regulated
SAMSN1	0.474798024	NA	up-regulated
GIMAP6	0.475659179	NA	up-regulated
SELE	0.485571765	NA	up-regulated
CXCL2	0.488521642	NA	up-regulated
BST2	0.492442397	NA	up-regulated
CX3CR1	0.495095466	NA	up-regulated
GBP5	0.49538969	NA	up-regulated
CCL14	0.499093657	NA	up-regulated
C14orf56	0.501474552	NA	up-regulated
FLJ21438	0.512063333	NA	up-regulated
FLJ22662	0.517501797	NA	up-regulated
ADH1A	0.523740336	NA	up-regulated
LILRB2	0.533985	NA	up-regulated
CFB	0.538195007	NA	up-regulated
MEOX2	0.540017753	NA	up-regulated
GIMAP4	0.541093544	NA	up-regulated
OGN	0.545099469	NA	up-regulated
GLI1	0.550371452	NA	up-regulated
ISG20	0.552504983	NA	up-regulated
AMICA1	0.553981094	NA	up-regulated
IFITM4P	0.556286808	NA	up-regulated
IBRDC2	0.580560047	NA	up-regulated
C1QC	0.584621633	NA	up-regulated
NGFR	0.592452269	NA	up-regulated
LOC402505	0.599830629	NA	down-regulated
CH25H	0.602649911	NA	up-regulated
CCL8	0.60408966	NA	up-regulated
LAPTM5	0.608015533	NA	up-regulated
FPR1	0.608193177	NA	up-regulated
CD28	0.609925153	NA	up-regulated
HCST	0.610595921	NA	up-regulated
VAMP8	0.620555273	NA	up-regulated
HLA-F	0.639251527	NA	up-regulated

ARID5A	0.645638403	NA	up-regulated
RARRES2	0.660114128	NA	up-regulated
MX1	0.670233587	NA	up-regulated
GBP1	0.678646258	NA	up-regulated
CPVL	0.683001201	NA	up-regulated
CTSS	0.683361982	NA	up-regulated
EMP3	0.683723622	NA	up-regulated
VAMP5	0.694977873	NA	up-regulated
C1R	0.703936227	NA	up-regulated
TAP1	0.705379673	NA	up-regulated
APOD	0.706745382	NA	up-regulated
FCER1A	0.714321059	NA	up-regulated
CCL13	0.71457483	NA	up-regulated
CEBPD	0.714994417	NA	up-regulated
STAT6	0.717810691	NA	up-regulated
GADD45B	0.721042791	NA	up-regulated
MGC29506	0.726462112	NA	up-regulated
GBP2	0.733215487	NA	up-regulated
IGF1	0.736175681	NA	up-regulated
GGTA1	0.738950421	NA	up-regulated
MFNG	0.76471774	NA	up-regulated
OMD	0.770134966	NA	up-regulated
NCF2	0.778930344	NA	up-regulated
PAMCI	0.786444924	NA	up-regulated
ARRDC2	0.787728312	NA	up-regulated
IL4R	0.788196832	NA	up-regulated
BATF	0.788307798	NA	up-regulated
ITPR3	0.790811215	NA	up-regulated
CXCL9	0.800842835	NA	up-regulated
ZC3H12D	0.803128113	NA	up-regulated
SELP	0.803593827	NA	up-regulated
CFHR3	0.822542025	NA	up-regulated
RTP4	0.828209078	NA	up-regulated
S100A8	0.832011034	NA	up-regulated
GZMB	0.832421329	NA	up-regulated
SQRDL	0.832605199	NA	up-regulated
MNDA	0.835821615	NA	up-regulated
IFITM1	0.836389482	NA	up-regulated
PLSCR1	0.854872383	NA	up-regulated
GGT5	0.858450557	NA	up-regulated
CFI	0.862293159	NA	up-regulated
LY96	0.867582192	NA	up-regulated
HSD11B1	0.870047031	NA	up-regulated
C1QB	0.870986453	NA	up-regulated

POU2AF1	0.883518324	NA	up-regulated
CFD	0.894453575	NA	up-regulated
ZC3H12A	0.898275359	NA	up-regulated
C7	0.900276887	NA	up-regulated
LOC642652	0.905518886	NA	up-regulated
CASP4	0.907819272	NA	up-regulated
MFAP5	0.918493137	NA	up-regulated
C10orf56	0.927808722	NA	up-regulated
TMEM154	0.930155976	NA	up-regulated
XAF1	0.936625917	NA	up-regulated
MS4A4A	0.941137164	NA	up-regulated
SP110	0.955143347	NA	up-regulated
RARRES3	0.957196072	NA	up-regulated
EPSTI1	0.962847207	NA	up-regulated
CASP5	0.971083123	NA	up-regulated
IGJ	0.974257346	NA	up-regulated
SLCO2A1	0.988124818	NA	up-regulated
COL8A2	0.988750713	NA	up-regulated
SUSD3	0.991797187	NA	up-regulated
GIMAP7	0.997668012	NA	up-regulated

TME: the tumor microenvironment.

Table S4. The gene ontology term enrichment of 280 common differentially expressed genes with p.adjust < 0.05

ONTOLOGY	ID	Description	p.adjust
BP	GO:0034341	response to interferon-gamma	6.07E-23
BP	GO:0071346	cellular response to interferon-gamma	7.32E-21
BP	GO:0042110	T cell activation	2.07E-18
BP	GO:0051249	regulation of lymphocyte activation	6.14E-17
BP	GO:0060326	cell chemotaxis	8.47E-17
BP	GO:0050900	leukocyte migration	1.09E-16
BP	GO:0050867	positive regulation of cell activation	1.84E-16
BP	GO:0007159	leukocyte cell-cell adhesion	1.07E-15
BP	GO:0002697	regulation of immune effector process	1.93E-15
BP	GO:0072676	lymphocyte migration	1.93E-15
BP	GO:0070661	leukocyte proliferation	1.93E-15
BP	GO:1990868	response to chemokine	1.93E-15
BP	GO:1990869	cellular response to chemokine	1.93E-15
BP	GO:0001819	positive regulation of cytokine production	1.93E-15
BP	GO:0002696	positive regulation of leukocyte activation	2.12E-15
BP	GO:0002460	adaptive immune response based on somatic recombination of immune receptors built from immunoglobulin superfamily domains	3.85E-15
BP	GO:0070098	chemokine-mediated signaling pathway	5.21E-15
BP	GO:0070663	regulation of leukocyte proliferation	8.99E-15
BP	GO:0030595	leukocyte chemotaxis	1.06E-14
BP	GO:0019882	antigen processing and presentation	1.24E-14
BP	GO:0051251	positive regulation of lymphocyte activation	2.55E-14
BP	GO:0030217	T cell differentiation	4.74E-14
BP	GO:0030098	lymphocyte differentiation	9.95E-14
BP	GO:0050870	positive regulation of T cell activation	9.95E-14
BP	GO:0006959	humoral immune response	1.17E-13
BP	GO:0060333	interferon-gamma-mediated signaling pathway	1.34E-13
BP	GO:0050670	regulation of lymphocyte proliferation	1.69E-13
BP	GO:0032944	regulation of mononuclear cell proliferation	1.81E-13
BP	GO:0050863	regulation of T cell activation	2.77E-13
BP	GO:1903039	positive regulation of leukocyte cell-cell adhesion	4.27E-13

BP	GO:0002449	lymphocyte mediated immunity	5.58E-13
BP	GO:0046651	lymphocyte proliferation	6.15E-13
BP	GO:0009615	response to virus	6.15E-13
BP	GO:0032943	mononuclear cell proliferation	6.88E-13
BP	GO:0046631	alpha-beta T cell activation	7.18E-13
BP	GO:0070665	positive regulation of leukocyte proliferation	8.00E-13
BP	GO:1903037	regulation of leukocyte cell-cell adhesion	8.36E-13
BP	GO:0022409	positive regulation of cell-cell adhesion	1.09E-12
BP	GO:0097529	myeloid leukocyte migration	1.54E-12
BP	GO:0045785	positive regulation of cell adhesion	1.85E-12
BP	GO:0050671	positive regulation of lymphocyte proliferation	2.86E-12
BP	GO:0048247	lymphocyte chemotaxis	2.86E-12
BP	GO:0032946	positive regulation of mononuclear cell proliferation	3.16E-12
BP	GO:0002548	monocyte chemotaxis	3.44E-12
BP	GO:0019884	antigen processing and presentation of exogenous antigen	8.60E-12
BP	GO:0002819	regulation of adaptive immune response	8.60E-12
BP	GO:0022407	regulation of cell-cell adhesion	1.08E-11
BP	GO:0071674	mononuclear cell migration	2.13E-11
BP	GO:0002685	regulation of leukocyte migration	3.26E-11
BP	GO:0002478	antigen processing and presentation of exogenous peptide antigen	4.07E-11
BP	GO:0042102	positive regulation of T cell proliferation	4.57E-11
BP	GO:0002683	negative regulation of immune system process	4.78E-11
BP	GO:0050852	T cell receptor signaling pathway	5.32E-11
BP	GO:1990266	neutrophil migration	8.32E-11
BP	GO:0097530	granulocyte migration	1.13E-10
BP	GO:0051607	defense response to virus	1.26E-10
BP	GO:0032103	positive regulation of response to external stimulus	1.29E-10
BP	GO:0050727	regulation of inflammatory response	1.36E-10
BP	GO:0048002	antigen processing and presentation of peptide antigen	1.38E-10
BP	GO:0071621	granulocyte chemotaxis	1.44E-10
BP	GO:0030593	neutrophil chemotaxis	1.50E-10
BP	GO:0050851	antigen receptor-mediated signaling pathway	5.32E-10
BP	GO:0002285	lymphocyte activation involved in immune response	5.95E-10

BP	GO:0002822	regulation of adaptive immune response based on somatic recombination of immune receptors built from immunoglobulin superfamily domains	1.73E-09
BP	GO:0042129	regulation of T cell proliferation	5.19E-09
BP	GO:0002699	positive regulation of immune effector process	1.08E-08
BP	GO:0016064	immunoglobulin mediated immune response	1.24E-08
BP	GO:0019724	B cell mediated immunity	1.52E-08
BP	GO:0019079	viral genome replication	1.65E-08
BP	GO:0046632	alpha-beta T cell differentiation	1.65E-08
BP	GO:0072678	T cell migration	1.85E-08
BP	GO:0002286	T cell activation involved in immune response	2.95E-08
BP	GO:0002687	positive regulation of leukocyte migration	2.96E-08
BP	GO:0006968	cellular defense response	3.68E-08
BP	GO:0050792	regulation of viral process	4.12E-08
BP	GO:0002429	immune response-activating cell surface receptor signaling pathway	4.87E-08
BP	GO:0050663	cytokine secretion	5.06E-08
BP	GO:0042098	T cell proliferation	5.06E-08
BP	GO:0032609	interferon-gamma production	6.01E-08
BP	GO:0002688	regulation of leukocyte chemotaxis	6.63E-08
BP	GO:0046634	regulation of alpha-beta T cell activation	6.66E-08
BP	GO:0042113	B cell activation	7.83E-08
BP	GO:0045069	regulation of viral genome replication	8.14E-08
BP	GO:0060337	type I interferon signaling pathway	8.14E-08
BP	GO:0071357	cellular response to type I interferon	8.14E-08
BP	GO:0045071	negative regulation of viral genome replication	9.56E-08
BP	GO:0043903	regulation of symbiosis, encompassing mutualism through parasitism	9.63E-08
BP	GO:0019886	antigen processing and presentation of exogenous peptide antigen via MHC class II	1.13E-07
BP	GO:0034340	response to type I interferon	1.26E-07
BP	GO:0043901	negative regulation of multi-organism process	1.29E-07
BP	GO:0002824	positive regulation of adaptive immune response based on somatic recombination of immune receptors built from immunoglobulin superfamily domains	1.38E-07
BP	GO:0002495	antigen processing and presentation of peptide antigen via MHC class II	1.54E-07
BP	GO:0002504	antigen processing and presentation of peptide or polysaccharide antigen via MHC class II	1.70E-07
BP	GO:0050777	negative regulation of immune response	1.86E-07

BP	GO:0032496	response to lipopolysaccharide	1.95E-07
BP	GO:0019883	antigen processing and presentation of endogenous antigen	1.95E-07
BP	GO:0002821	positive regulation of adaptive immune response	2.28E-07
BP	GO:0043900	regulation of multi-organism process	2.41E-07
BP	GO:0050707	regulation of cytokine secretion	2.70E-07
BP	GO:1902105	regulation of leukocyte differentiation	2.85E-07
BP	GO:0002690	positive regulation of leukocyte chemotaxis	3.21E-07
BP	GO:0002292	T cell differentiation involved in immune response	3.38E-07
BP	GO:0002237	response to molecule of bacterial origin	3.49E-07
BP	GO:0002526	acute inflammatory response	4.99E-07
BP	GO:0045580	regulation of T cell differentiation	5.77E-07
BP	GO:0055074	calcium ion homeostasis	7.31E-07
BP	GO:0045619	regulation of lymphocyte differentiation	7.59E-07
BP	GO:0002703	regulation of leukocyte mediated immunity	9.66E-07
BP	GO:0035456	response to interferon-beta	1.12E-06
BP	GO:0048525	negative regulation of viral process	1.16E-06
BP	GO:1903900	regulation of viral life cycle	1.26E-06
BP	GO:0007249	I-kappaB kinase/NF-kappaB signaling	1.31E-06
BP	GO:0042088	T-helper 1 type immune response	1.37E-06
BP	GO:0032611	interleukin-1 beta production	1.37E-06
BP	GO:0032649	regulation of interferon-gamma production	1.37E-06
BP	GO:0072503	cellular divalent inorganic cation homeostasis	1.54E-06
BP	GO:0002287	alpha-beta T cell activation involved in immune response	1.56E-06
BP	GO:0002293	alpha-beta T cell differentiation involved in immune response	1.56E-06
BP	GO:0006874	cellular calcium ion homeostasis	1.77E-06
BP	GO:0046635	positive regulation of alpha-beta T cell activation	1.77E-06
BP	GO:0042119	neutrophil activation	1.77E-06
BP	GO:0002791	regulation of peptide secretion	1.89E-06
BP	GO:0034612	response to tumor necrosis factor	1.90E-06
BP	GO:2000106	regulation of leukocyte apoptotic process	1.97E-06
BP	GO:2000401	regulation of lymphocyte migration	1.97E-06
BP	GO:0050920	regulation of chemotaxis	2.27E-06
BP	GO:0001909	leukocyte mediated cytotoxicity	2.27E-06

BP	GO:1903901	negative regulation of viral life cycle	2.41E-06
BP	GO:0002705	positive regulation of leukocyte mediated immunity	2.45E-06
BP	GO:0045058	T cell selection	2.45E-06
BP	GO:2000107	negative regulation of leukocyte apoptotic process	2.45E-06
BP	GO:0032680	regulation of tumor necrosis factor production	2.46E-06
BP	GO:0050921	positive regulation of chemotaxis	2.84E-06
BP	GO:0098883	synapse pruning	2.84E-06
BP	GO:0032640	tumor necrosis factor production	2.97E-06
BP	GO:1903555	regulation of tumor necrosis factor superfamily cytokine production	2.97E-06
BP	GO:0071356	cellular response to tumor necrosis factor	3.32E-06
BP	GO:0045061	thymic T cell selection	3.58E-06
BP	GO:0050715	positive regulation of cytokine secretion	3.75E-06
BP	GO:0032651	regulation of interleukin-1 beta production	3.82E-06
BP	GO:0043312	neutrophil degranulation	4.02E-06
BP	GO:0001906	cell killing	4.02E-06
BP	GO:0071706	tumor necrosis factor superfamily cytokine production	4.02E-06
BP	GO:0032612	interleukin-1 production	4.21E-06
BP	GO:0002283	neutrophil activation involved in immune response	4.38E-06
BP	GO:0042092	type 2 immune response	4.41E-06
BP	GO:0035710	CD4-positive, alpha-beta T cell activation	4.49E-06
BP	GO:0071222	cellular response to lipopolysaccharide	6.10E-06
BP	GO:0002446	neutrophil mediated immunity	6.24E-06
BP	GO:0002698	negative regulation of immune effector process	6.25E-06
BP	GO:2000403	positive regulation of lymphocyte migration	6.39E-06
BP	GO:0070555	response to interleukin-1	6.69E-06
BP	GO:0002706	regulation of lymphocyte mediated immunity	7.28E-06
BP	GO:0002407	dendritic cell chemotaxis	7.71E-06
BP	GO:0001912	positive regulation of leukocyte mediated cytotoxicity	8.51E-06
BP	GO:0071219	cellular response to molecule of bacterial origin	8.71E-06
BP	GO:0050729	positive regulation of inflammatory response	9.45E-06
BP	GO:0050708	regulation of protein secretion	9.56E-06
BP	GO:0050901	leukocyte tethering or rolling	9.66E-06
BP	GO:0090025	regulation of monocyte chemotaxis	9.66E-06

BP	GO:0001910	regulation of leukocyte mediated cytotoxicity	1.00E-05
BP	GO:1903706	regulation of hemopoiesis	1.03E-05
BP	GO:0042093	T-helper cell differentiation	1.07E-05
BP	GO:0002440	production of molecular mediator of immune response	1.16E-05
BP	GO:0032652	regulation of interleukin-1 production	1.16E-05
BP	GO:0002793	positive regulation of peptide secretion	1.26E-05
BP	GO:0071887	leukocyte apoptotic process	1.26E-05
BP	GO:0002673	regulation of acute inflammatory response	1.33E-05
BP	GO:0002294	CD4-positive, alpha-beta T cell differentiation involved in immune response	1.35E-05
BP	GO:0002708	positive regulation of lymphocyte mediated immunity	1.35E-05
BP	GO:0002483	antigen processing and presentation of endogenous peptide antigen	1.38E-05
BP	GO:0019885	antigen processing and presentation of endogenous peptide antigen via MHC class I	1.38E-05
BP	GO:0019058	viral life cycle	1.42E-05
BP	GO:0036336	dendritic cell migration	1.45E-05
BP	GO:0002920	regulation of humoral immune response	1.63E-05
BP	GO:2000404	regulation of T cell migration	1.90E-05
BP	GO:0002475	antigen processing and presentation via MHC class Ib	1.93E-05
BP	GO:0002820	negative regulation of adaptive immune response	2.20E-05
BP	GO:0050866	negative regulation of cell activation	2.23E-05
BP	GO:0032729	positive regulation of interferon-gamma production	2.37E-05
BP	GO:0050714	positive regulation of protein secretion	2.37E-05
BP	GO:0071675	regulation of mononuclear cell migration	2.51E-05
BP	GO:0071216	cellular response to biotic stimulus	2.67E-05
BP	GO:0046640	regulation of alpha-beta T cell proliferation	2.67E-05
BP	GO:0032602	chemokine production	2.70E-05
BP	GO:0002573	myeloid leukocyte differentiation	2.83E-05
BP	GO:0030449	regulation of complement activation	2.84E-05
BP	GO:0051047	positive regulation of secretion	2.97E-05
BP	GO:2000257	regulation of protein activation cascade	3.04E-05
BP	GO:2000406	positive regulation of T cell migration	3.16E-05
BP	GO:0031343	positive regulation of cell killing	3.16E-05
BP	GO:0002695	negative regulation of leukocyte activation	3.23E-05
BP	GO:0090026	positive regulation of monocyte chemotaxis	3.40E-05

BP	GO:0051250	negative regulation of lymphocyte activation	3.43E-05
BP	GO:0001913	T cell mediated cytotoxicity	3.64E-05
BP	GO:0071347	cellular response to interleukin-1	4.00E-05
BP	GO:1903532	positive regulation of secretion by cell	4.27E-05
BP	GO:0042116	macrophage activation	4.38E-05
BP	GO:0046641	positive regulation of alpha-beta T cell proliferation	4.40E-05
BP	GO:0001914	regulation of T cell mediated cytotoxicity	4.40E-05
BP	GO:0032633	interleukin-4 production	4.40E-05
BP	GO:0046633	alpha-beta T cell proliferation	4.40E-05
BP	GO:0019730	antimicrobial humoral response	4.49E-05
BP	GO:0032755	positive regulation of interleukin-6 production	4.64E-05
BP	GO:0043547	positive regulation of GTPase activity	5.06E-05
BP	GO:0043368	positive T cell selection	5.19E-05
BP	GO:0031341	regulation of cell killing	5.43E-05
BP	GO:0016485	protein processing	5.48E-05
BP	GO:0043367	CD4-positive, alpha-beta T cell differentiation	5.53E-05
BP	GO:0002577	regulation of antigen processing and presentation	5.54E-05
BP	GO:0001818	negative regulation of cytokine production	6.90E-05
BP	GO:0030099	myeloid cell differentiation	7.09E-05
BP	GO:0048245	eosinophil chemotaxis	7.10E-05
BP	GO:0042590	antigen processing and presentation of exogenous peptide antigen via MHC class I	8.86E-05
BP	GO:0002604	regulation of dendritic cell antigen processing and presentation	0.000102131
BP	GO:0045060	negative thymic T cell selection	0.000102131
BP	GO:0032722	positive regulation of chemokine production	0.000104088
BP	GO:0097696	STAT cascade	0.00010589
BP	GO:0032642	regulation of chemokine production	0.000114568
BP	GO:0043122	regulation of I-kappaB kinase/NF-kappaB signaling	0.000123937
BP	GO:0043087	regulation of GTPase activity	0.000128133
BP	GO:0002700	regulation of production of molecular mediator of immune response	0.000132001
BP	GO:0042832	defense response to protozoan	0.00013681
BP	GO:0071677	positive regulation of mononuclear cell migration	0.00013681
BP	GO:0045123	cellular extravasation	0.000141129
BP	GO:0002468	dendritic cell antigen processing and presentation	0.000143091

BP	GO:0002863	positive regulation of inflammatory response to antigenic stimulus	0.000143091
BP	GO:0042340	keratan sulfate catabolic process	0.000143091
BP	GO:0043383	negative T cell selection	0.000143091
BP	GO:0070371	ERK1 and ERK2 cascade	0.000144138
BP	GO:0007204	positive regulation of cytosolic calcium ion concentration	0.000154289
BP	GO:0032760	positive regulation of tumor necrosis factor production	0.000154289
BP	GO:0001562	response to protozoan	0.000161364
BP	GO:0002335	mature B cell differentiation	0.000161364
BP	GO:0072677	eosinophil migration	0.000161364
BP	GO:0006956	complement activation	0.000161413
BP	GO:0042035	regulation of cytokine biosynthetic process	0.00016444
BP	GO:1902107	positive regulation of leukocyte differentiation	0.000168071
BP	GO:1903557	positive regulation of tumor necrosis factor superfamily cytokine production	0.000177609
BP	GO:0001916	positive regulation of T cell mediated cytotoxicity	0.000193649
BP	GO:0045059	positive thymic T cell selection	0.000194483
BP	GO:0070613	regulation of protein processing	0.000205122
BP	GO:0061756	leukocyte adhesion to vascular endothelial cell	0.000208125
BP	GO:0070374	positive regulation of ERK1 and ERK2 cascade	0.000208899
BP	GO:1904951	positive regulation of establishment of protein localization	0.000213457
BP	GO:1903317	regulation of protein maturation	0.000223758
BP	GO:0002825	regulation of T-helper 1 type immune response	0.00022676
BP	GO:0032673	regulation of interleukin-4 production	0.00022676
BP	GO:1901623	regulation of lymphocyte chemotaxis	0.00022676
BP	GO:0002455	humoral immune response mediated by circulating immunoglobulin	0.000228542
BP	GO:0043123	positive regulation of I-kappaB kinase/NF-kappaB signaling	0.000230809
BP	GO:0042108	positive regulation of cytokine biosynthetic process	0.000235808
BP	GO:0050864	regulation of B cell activation	0.000240918
BP	GO:0030336	negative regulation of cell migration	0.000241357
BP	GO:0051222	positive regulation of protein transport	0.000242046
BP	GO:0032675	regulation of interleukin-6 production	0.000250384
BP	GO:0010818	T cell chemotaxis	0.000264767
BP	GO:0042089	cytokine biosynthetic process	0.000278454
BP	GO:0045089	positive regulation of innate immune response	0.000284831

BP	GO:0002702	positive regulation of production of molecular mediator of immune response	0.000285485
BP	GO:0042100	B cell proliferation	0.000285485
BP	GO:0042107	cytokine metabolic process	0.000292509
BP	GO:0002709	regulation of T cell mediated immunity	0.000300638
BP	GO:0033077	T cell differentiation in thymus	0.000300638
BP	GO:0007259	JAK-STAT cascade	0.000302352
BP	GO:0002474	antigen processing and presentation of peptide antigen via MHC class I	0.000302352
BP	GO:0060142	regulation of syncytium formation by plasma membrane fusion	0.000304842
BP	GO:0001774	microglial cell activation	0.000313571
BP	GO:0002269	leukocyte activation involved in inflammatory response	0.000313571
BP	GO:0090197	positive regulation of chemokine secretion	0.000324984
BP	GO:0002828	regulation of type 2 immune response	0.000356781
BP	GO:2000146	negative regulation of cell motility	0.000374813
BP	GO:0002312	B cell activation involved in immune response	0.00038288
BP	GO:0032635	interleukin-6 production	0.000383926
BP	GO:0006957	complement activation, alternative pathway	0.000421293
BP	GO:0072376	protein activation cascade	0.000434549
BP	GO:0051604	protein maturation	0.000437262
BP	GO:0002479	antigen processing and presentation of exogenous peptide antigen via MHC class I, TAP-dependent	0.000446891
BP	GO:0150076	neuroinflammatory response	0.000446891
BP	GO:0051480	regulation of cytosolic calcium ion concentration	0.00047189
BP	GO:0032814	regulation of natural killer cell activation	0.00047611
BP	GO:0032731	positive regulation of interleukin-1 beta production	0.00047611
BP	GO:0050702	interleukin-1 beta secretion	0.000525771
BP	GO:0070228	regulation of lymphocyte apoptotic process	0.000525771
BP	GO:1905517	macrophage migration	0.000525771
BP	GO:0002456	T cell mediated immunity	0.000573849
BP	GO:0045088	regulation of innate immune response	0.000573849
BP	GO:0070206	protein trimerization	0.000579309
BP	GO:0006958	complement activation, classical pathway	0.000582434
BP	GO:0044273	sulfur compound catabolic process	0.000639116
BP	GO:0090196	regulation of chemokine secretion	0.000656424

BP	GO:0031295	T cell costimulation	0.000703595
BP	GO:0042742	defense response to bacterium	0.000733074
BP	GO:0031294	lymphocyte costimulation	0.00077299
BP	GO:0002313	mature B cell differentiation involved in immune response	0.000807395
BP	GO:0045063	T-helper 1 cell differentiation	0.000807395
BP	GO:0045582	positive regulation of T cell differentiation	0.000807395
BP	GO:0061900	glial cell activation	0.000841863
BP	GO:0032732	positive regulation of interleukin-1 production	0.000924432
BP	GO:0032816	positive regulation of natural killer cell activation	0.000985372
BP	GO:0090195	chemokine secretion	0.000985372
BP	GO:0051271	negative regulation of cellular component movement	0.000992458
BP	GO:0006919	activation of cysteine-type endopeptidase activity involved in apoptotic process	0.000992458
BP	GO:0070372	regulation of ERK1 and ERK2 cascade	0.001038001
BP	GO:0014068	positive regulation of phosphatidylinositol 3-kinase signaling	0.001061031
BP	GO:0030888	regulation of B cell proliferation	0.001086826
BP	GO:0050701	interleukin-1 secretion	0.001086826
BP	GO:1903708	positive regulation of hemopoiesis	0.001098894
BP	GO:1901739	regulation of myoblast fusion	0.001174193
BP	GO:0019932	second-messenger-mediated signaling	0.001269527
BP	GO:0046637	regulation of alpha-beta T cell differentiation	0.001285994
BP	GO:0002823	negative regulation of adaptive immune response based on somatic recombination of immune receptors built from immunoglobulin superfamily domains	0.001293336
BP	GO:0040013	negative regulation of locomotion	0.001344114
BP	GO:1904894	positive regulation of STAT cascade	0.001466218
BP	GO:0002377	immunoglobulin production	0.001521227
BP	GO:0002639	positive regulation of immunoglobulin production	0.001612758
BP	GO:0097028	dendritic cell differentiation	0.001612758
BP	GO:0032753	positive regulation of interleukin-4 production	0.001651308
BP	GO:0045621	positive regulation of lymphocyte differentiation	0.001651308
BP	GO:0002861	regulation of inflammatory response to antigenic stimulus	0.001954998
BP	GO:0071622	regulation of granulocyte chemotaxis	0.001992667
BP	GO:0002291	T cell activation via T cell receptor contact with antigen bound to MHC molecule on antigen presenting cell	0.00202024

BP	GO:0072610	interleukin-12 secretion	0.00202024
BP	GO:0097048	dendritic cell apoptotic process	0.00202024
BP	GO:2000668	regulation of dendritic cell apoptotic process	0.00202024
BP	GO:0070227	lymphocyte apoptotic process	0.002034578
BP	GO:0050706	regulation of interleukin-1 beta secretion	0.002412325
BP	GO:0060443	mammary gland morphogenesis	0.002412325
BP	GO:1901342	regulation of vasculature development	0.002539803
BP	GO:0002532	production of molecular mediator involved in inflammatory response	0.002539803
BP	GO:0002367	cytokine production involved in immune response	0.002640458
BP	GO:0035747	natural killer cell chemotaxis	0.002694344
BP	GO:0061844	antimicrobial humoral immune response mediated by antimicrobial peptide	0.002714208
BP	GO:0002711	positive regulation of T cell mediated immunity	0.002900232
BP	GO:0060143	positive regulation of syncytium formation by plasma membrane fusion	0.002995479
BP	GO:0045765	regulation of angiogenesis	0.002999414
BP	GO:0002686	negative regulation of leukocyte migration	0.003159598
BP	GO:0002704	negative regulation of leukocyte mediated immunity	0.003159598
BP	GO:0018146	keratan sulfate biosynthetic process	0.003420036
BP	GO:0048524	positive regulation of viral process	0.003444757
BP	GO:0019722	calcium-mediated signaling	0.003732836
BP	GO:0070664	negative regulation of leukocyte proliferation	0.003787452
BP	GO:0070229	negative regulation of lymphocyte apoptotic process	0.00388301
BP	GO:1904892	regulation of STAT cascade	0.004371381
BP	GO:0045954	positive regulation of natural killer cell mediated cytotoxicity	0.004374266
BP	GO:0046596	regulation of viral entry into host cell	0.004374266
BP	GO:0002864	regulation of acute inflammatory response to antigenic stimulus	0.004374266
BP	GO:0035589	G protein-coupled purinergic nucleotide receptor signaling pathway	0.004374266
BP	GO:0050704	regulation of interleukin-1 secretion	0.004423328
BP	GO:0032655	regulation of interleukin-12 production	0.004798409
BP	GO:0032663	regulation of interleukin-2 production	0.004798409
BP	GO:2001056	positive regulation of cysteine-type endopeptidase activity	0.004888307
BP	GO:0002888	positive regulation of myeloid leukocyte mediated immunity	0.004888307
BP	GO:0010543	regulation of platelet activation	0.004888307
BP	GO:0008360	regulation of cell shape	0.005067569

BP	GO:0002886	regulation of myeloid leukocyte mediated immunity	0.005152055
BP	GO:0002523	leukocyte migration involved in inflammatory response	0.005362236
BP	GO:0045064	T-helper 2 cell differentiation	0.005362236
BP	GO:0002718	regulation of cytokine production involved in immune response	0.005368309
BP	GO:0070207	protein homotrimerization	0.005440394
BP	GO:0000768	syncytium formation by plasma membrane fusion	0.005492579
BP	GO:0032615	interleukin-12 production	0.005492579
BP	GO:0140253	cell-cell fusion	0.005492579
BP	GO:0030168	platelet activation	0.005631342
BP	GO:0060760	positive regulation of response to cytokine stimulus	0.005927853
BP	GO:0030101	natural killer cell activation	0.005966582
BP	GO:0002717	positive regulation of natural killer cell mediated immunity	0.005981406
BP	GO:0042339	keratan sulfate metabolic process	0.005981406
BP	GO:0070232	regulation of T cell apoptotic process	0.005981406
BP	GO:0030198	extracellular matrix organization	0.006087804
BP	GO:0006949	syncytium formation	0.006318379
BP	GO:0030852	regulation of granulocyte differentiation	0.00639157
BP	GO:0042533	tumor necrosis factor biosynthetic process	0.006606001
BP	GO:0042534	regulation of tumor necrosis factor biosynthetic process	0.006606001
BP	GO:0070670	response to interleukin-4	0.006606001
BP	GO:0031348	negative regulation of defense response	0.006888744
BP	GO:2000116	regulation of cysteine-type endopeptidase activity	0.006888744
BP	GO:0046427	positive regulation of JAK-STAT cascade	0.006923867
BP	GO:0042267	natural killer cell mediated cytotoxicity	0.00720644
BP	GO:0045576	mast cell activation	0.00720644
BP	GO:0032735	positive regulation of interleukin-12 production	0.007270018
BP	GO:0014066	regulation of phosphatidylinositol 3-kinase signaling	0.007411294
BP	GO:0002830	positive regulation of type 2 immune response	0.007551094
BP	GO:0061158	3'-UTR-mediated mRNA destabilization	0.007551094
BP	GO:0006027	glycosaminoglycan catabolic process	0.007630907
BP	GO:0090303	positive regulation of wound healing	0.007630907
BP	GO:2000514	regulation of CD4-positive, alpha-beta T cell activation	0.007630907
BP	GO:1902622	regulation of neutrophil migration	0.007933217

BP	GO:2000516	positive regulation of CD4-positive, alpha-beta T cell activation	0.007933217
BP	GO:0007162	negative regulation of cell adhesion	0.008066128
BP	GO:0032623	interleukin-2 production	0.008133348
BP	GO:0042063	gliogenesis	0.008252027
BP	GO:0002637	regulation of immunoglobulin production	0.008653249
BP	GO:0002714	positive regulation of B cell mediated immunity	0.008653249
BP	GO:0002891	positive regulation of immunoglobulin mediated immune response	0.008653249
BP	GO:2000249	regulation of actin cytoskeleton reorganization	0.008653249
BP	GO:0010819	regulation of T cell chemotaxis	0.008765874
BP	GO:1903975	regulation of glial cell migration	0.008765874
BP	GO:1903038	negative regulation of leukocyte cell-cell adhesion	0.00894296
BP	GO:0002228	natural killer cell mediated immunity	0.009158628
BP	GO:0046782	regulation of viral transcription	0.009158628
BP	GO:0048246	macrophage chemotaxis	0.00943501
BP	GO:0006026	aminoglycan catabolic process	0.009774124
BP	GO:0043280	positive regulation of cysteine-type endopeptidase activity involved in apoptotic process	0.010093806
BP	GO:0001911	negative regulation of leukocyte mediated cytotoxicity	0.010217679
BP	GO:0070233	negative regulation of T cell apoptotic process	0.010217679
BP	GO:0050856	regulation of T cell receptor signaling pathway	0.010258783
BP	GO:1905521	regulation of macrophage migration	0.010258783
BP	GO:0043281	regulation of cysteine-type endopeptidase activity involved in apoptotic process	0.011412186
BP	GO:0002544	chronic inflammatory response	0.011819062
BP	GO:0045076	regulation of interleukin-2 biosynthetic process	0.011819062
BP	GO:0045414	regulation of interleukin-8 biosynthetic process	0.011819062
BP	GO:1901741	positive regulation of myoblast fusion	0.011819062
BP	GO:0050830	defense response to Gram-positive bacterium	0.012150372
BP	GO:0046425	regulation of JAK-STAT cascade	0.012150372
BP	GO:0007520	myoblast fusion	0.012150372
BP	GO:0010950	positive regulation of endopeptidase activity	0.013015784
BP	GO:0010559	regulation of glycoprotein biosynthetic process	0.013236359
BP	GO:0002827	positive regulation of T-helper 1 type immune response	0.01351623
BP	GO:0010560	positive regulation of glycoprotein biosynthetic process	0.01351623
BP	GO:0042228	interleukin-8 biosynthetic process	0.01351623

BP	GO:0042531	positive regulation of tyrosine phosphorylation of STAT protein	0.013855022
BP	GO:0048015	phosphatidylinositol-mediated signaling	0.014249265
BP	GO:0018108	peptidyl-tyrosine phosphorylation	0.014743481
BP	GO:0042136	neurotransmitter biosynthetic process	0.015187679
BP	GO:0002438	acute inflammatory response to antigenic stimulus	0.015269147
BP	GO:0035455	response to interferon-alpha	0.015269147
BP	GO:0035590	purinergic nucleotide receptor signaling pathway	0.015269147
BP	GO:0140131	positive regulation of lymphocyte chemotaxis	0.015269147
BP	GO:0030225	macrophage differentiation	0.015269147
BP	GO:0042269	regulation of natural killer cell mediated cytotoxicity	0.015269147
BP	GO:0032945	negative regulation of mononuclear cell proliferation	0.015269147
BP	GO:0050672	negative regulation of lymphocyte proliferation	0.015269147
BP	GO:0048017	inositol lipid-mediated signaling	0.01538037
BP	GO:0018212	peptidyl-tyrosine modification	0.01538037
BP	GO:0032677	regulation of interleukin-8 production	0.016064288
BP	GO:1903036	positive regulation of response to wounding	0.016064288
BP	GO:0045581	negative regulation of T cell differentiation	0.016334089
BP	GO:1904018	positive regulation of vasculature development	0.016824976
BP	GO:0031342	negative regulation of cell killing	0.017075979
BP	GO:0032656	regulation of interleukin-13 production	0.017075979
BP	GO:0042094	interleukin-2 biosynthetic process	0.017075979
BP	GO:0043902	positive regulation of multi-organism process	0.017218212
BP	GO:0043062	extracellular structure organization	0.017218212
BP	GO:0002437	inflammatory response to antigenic stimulus	0.017360938
BP	GO:0002715	regulation of natural killer cell mediated immunity	0.017360938
BP	GO:0043300	regulation of leukocyte degranulation	0.017360938
BP	GO:0014065	phosphatidylinositol 3-kinase signaling	0.017475626
BP	GO:0052548	regulation of endopeptidase activity	0.018096218
BP	GO:0032481	positive regulation of type I interferon production	0.018527801
BP	GO:0051851	modification by host of symbiont morphology or physiology	0.018527801
BP	GO:0046638	positive regulation of alpha-beta T cell differentiation	0.018544572
BP	GO:0070231	T cell apoptotic process	0.018544572
BP	GO:0050868	negative regulation of T cell activation	0.018753028

BP	GO:0009595	detection of biotic stimulus	0.018861303
BP	GO:0010447	response to acidic pH	0.018861303
BP	GO:0051235	maintenance of location	0.019527712
BP	GO:0032648	regulation of interferon-beta production	0.019774136
BP	GO:0072604	interleukin-6 secretion	0.019774136
BP	GO:0051048	negative regulation of secretion	0.020127575
BP	GO:0072593	reactive oxygen species metabolic process	0.020127575
BP	GO:0006690	icosanoid metabolic process	0.020127575
BP	GO:0042133	neurotransmitter metabolic process	0.020355246
BP	GO:0032147	activation of protein kinase activity	0.02060468
BP	GO:0032897	negative regulation of viral transcription	0.020693837
BP	GO:0035588	G protein-coupled purinergic receptor signaling pathway	0.020693837
BP	GO:0043302	positive regulation of leukocyte degranulation	0.020693837
BP	GO:0090023	positive regulation of neutrophil chemotaxis	0.020693837
BP	GO:1903020	positive regulation of glycoprotein metabolic process	0.020693837
BP	GO:0071902	positive regulation of protein serine/threonine kinase activity	0.020693837
BP	GO:0008347	glial cell migration	0.020693837
BP	GO:0032757	positive regulation of interleukin-8 production	0.020693837
BP	GO:1903018	regulation of glycoprotein metabolic process	0.020693837
BP	GO:1903707	negative regulation of hemopoiesis	0.021367028
BP	GO:0010952	positive regulation of peptidase activity	0.021396535
BP	GO:0032608	interferon-beta production	0.022136937
BP	GO:0032970	regulation of actin filament-based process	0.022523113
BP	GO:0002507	tolerance induction	0.022728536
BP	GO:0032616	interleukin-13 production	0.022728536
BP	GO:0034110	regulation of homotypic cell-cell adhesion	0.022728536
BP	GO:0032637	interleukin-8 production	0.022728536
BP	GO:0046209	nitric oxide metabolic process	0.022728536
BP	GO:0051702	interaction with symbiont	0.022728536
BP	GO:0002712	regulation of B cell mediated immunity	0.023300887
BP	GO:0002889	regulation of immunoglobulin mediated immune response	0.023300887
BP	GO:0048260	positive regulation of receptor-mediated endocytosis	0.023300887
BP	GO:0042509	regulation of tyrosine phosphorylation of STAT protein	0.023748855

BP	GO:0032956	regulation of actin cytoskeleton organization	0.024488605
BP	GO:0022612	gland morphogenesis	0.02449905
BP	GO:0032653	regulation of interleukin-10 production	0.024796388
BP	GO:0030278	regulation of ossification	0.024816154
BP	GO:0010758	regulation of macrophage chemotaxis	0.024819507
BP	GO:0030194	positive regulation of blood coagulation	0.024819507
BP	GO:0071624	positive regulation of granulocyte chemotaxis	0.024819507
BP	GO:1900048	positive regulation of hemostasis	0.024819507
BP	GO:0046718	viral entry into host cell	0.025155735
BP	GO:0045766	positive regulation of angiogenesis	0.025251138
BP	GO:2001057	reactive nitrogen species metabolic process	0.025719739
BP	GO:0007260	tyrosine phosphorylation of STAT protein	0.026982512
BP	GO:0052547	regulation of peptidase activity	0.027327193
BP	GO:0050820	positive regulation of coagulation	0.02733718
BP	GO:0002720	positive regulation of cytokine production involved in immune response	0.02762423
BP	GO:0002763	positive regulation of myeloid leukocyte differentiation	0.02762423
BP	GO:0030199	collagen fibril organization	0.02762423
BP	GO:0051209	release of sequestered calcium ion into cytosol	0.027842843
BP	GO:0051701	interaction with host	0.02866317
BP	GO:0050829	defense response to Gram-negative bacterium	0.02904439
BP	GO:0032613	interleukin-10 production	0.02904439
BP	GO:0042306	regulation of protein import into nucleus	0.02904439
BP	GO:0043030	regulation of macrophage activation	0.02904439
BP	GO:0045620	negative regulation of lymphocyte differentiation	0.02904439
BP	GO:0046456	icosanoid biosynthetic process	0.02904439
BP	GO:0009612	response to mechanical stimulus	0.029085517
BP	GO:0032479	regulation of type I interferon production	0.029457968
BP	GO:0051283	negative regulation of sequestering of calcium ion	0.029457968
BP	GO:0001773	myeloid dendritic cell activation	0.029457968
BP	GO:1903531	negative regulation of secretion by cell	0.029685355
BP	GO:0050806	positive regulation of synaptic transmission	0.030355972
BP	GO:0002381	immunoglobulin production involved in immunoglobulin mediated immune response	0.030502424
BP	GO:0098586	cellular response to virus	0.030502424

BP	GO:0048660	regulation of smooth muscle cell proliferation	0.03116134
BP	GO:0032606	type I interferon production	0.031350158
BP	GO:0051282	regulation of sequestering of calcium ion	0.031350158
BP	GO:0044788	modulation by host of viral process	0.031940145
BP	GO:0072538	T-helper 17 type immune response	0.031940145
BP	GO:1902624	positive regulation of neutrophil migration	0.031940145
BP	GO:0048659	smooth muscle cell proliferation	0.032815266
BP	GO:0031663	lipopolysaccharide-mediated signaling pathway	0.033975212
BP	GO:1904589	regulation of protein import	0.033975212
BP	GO:0045862	positive regulation of proteolysis	0.034254369
BP	GO:0030183	B cell differentiation	0.03438316
BP	GO:0051208	sequestering of calcium ion	0.03438316
BP	GO:0016525	negative regulation of angiogenesis	0.03438316
BP	GO:0032728	positive regulation of interferon-beta production	0.03438316
BP	GO:0032743	positive regulation of interleukin-2 production	0.03438316
BP	GO:0050869	negative regulation of B cell activation	0.03438316
BP	GO:0061157	mRNA destabilization	0.03438316
BP	GO:0090022	regulation of neutrophil chemotaxis	0.03438316
BP	GO:0045824	negative regulation of innate immune response	0.035427831
BP	GO:2000181	negative regulation of blood vessel morphogenesis	0.036231441
BP	GO:0001768	establishment of T cell polarity	0.036558773
BP	GO:0032621	interleukin-18 production	0.036558773
BP	GO:0045625	regulation of T-helper 1 cell differentiation	0.036558773
BP	GO:0060033	anatomical structure regression	0.036558773
BP	GO:1903265	positive regulation of tumor necrosis factor-mediated signaling pathway	0.036558773
BP	GO:2000425	regulation of apoptotic cell clearance	0.036558773
BP	GO:0002675	positive regulation of acute inflammatory response	0.036965353
BP	GO:0006691	leukotriene metabolic process	0.036965353
BP	GO:0030260	entry into host cell	0.036965353
BP	GO:0044409	entry into host	0.036965353
BP	GO:0051806	entry into cell of other organism involved in symbiotic interaction	0.036965353
BP	GO:0051828	entry into other organism involved in symbiotic interaction	0.036965353
BP	GO:0006909	phagocytosis	0.036965353

BP	GO:0043491	protein kinase B signaling	0.03698581
BP	GO:1903902	positive regulation of viral life cycle	0.038700473
BP	GO:0035587	purinergic receptor signaling pathway	0.039698602
BP	GO:0050779	RNA destabilization	0.039698602
BP	GO:0071353	cellular response to interleukin-4	0.039698602
BP	GO:2000778	positive regulation of interleukin-6 secretion	0.039698602
BP	GO:0002562	somatic diversification of immune receptors via germline recombination within a single locus	0.040463944
BP	GO:0010830	regulation of myotube differentiation	0.040463944
BP	GO:0016444	somatic cell DNA recombination	0.040463944
BP	GO:0022408	negative regulation of cell-cell adhesion	0.041526486
BP	GO:0030851	granulocyte differentiation	0.042156977
BP	GO:0032691	negative regulation of interleukin-1 beta production	0.042156977
BP	GO:0050718	positive regulation of interleukin-1 beta secretion	0.042156977
BP	GO:0060603	mammary gland duct morphogenesis	0.042156977
BP	GO:0001767	establishment of lymphocyte polarity	0.042156977
BP	GO:0002517	T cell tolerance induction	0.042156977
BP	GO:0032490	detection of molecule of bacterial origin	0.042156977
BP	GO:0032908	regulation of transforming growth factor beta1 production	0.042156977
BP	GO:0044793	negative regulation by host of viral process	0.042156977
BP	GO:0051024	positive regulation of immunoglobulin secretion	0.042156977
BP	GO:0072683	T cell extravasation	0.042156977
BP	GO:0002831	regulation of response to biotic stimulus	0.042218402
BP	GO:0097553	calcium ion transmembrane import into cytosol	0.043587705
BP	GO:0045669	positive regulation of osteoblast differentiation	0.04403668
BP	GO:0006816	calcium ion transport	0.044200281
BP	GO:0050673	epithelial cell proliferation	0.044200281
BP	GO:0010574	regulation of vascular endothelial growth factor production	0.045329632
BP	GO:0031532	actin cytoskeleton reorganization	0.045330813
BP	GO:0048661	positive regulation of smooth muscle cell proliferation	0.045330813
BP	GO:0050854	regulation of antigen receptor-mediated signaling pathway	0.045940846
BP	GO:0050871	positive regulation of B cell activation	0.045940846
BP	GO:1901343	negative regulation of vasculature development	0.047307135
BP	GO:0030879	mammary gland development	0.047307135

BP	GO:0002701	negative regulation of production of molecular mediator of immune response	0.048162828
BP	GO:0045070	positive regulation of viral genome replication	0.048162828
BP	GO:0045622	regulation of T-helper cell differentiation	0.048162828
BP	GO:1902106	negative regulation of leukocyte differentiation	0.048162828
BP	GO:0006069	ethanol oxidation	0.048162828
BP	GO:0032905	transforming growth factor beta1 production	0.048162828
BP	GO:0036005	response to macrophage colony-stimulating factor	0.048162828
BP	GO:0036006	cellular response to macrophage colony-stimulating factor stimulus	0.048162828
BP	GO:0042308	negative regulation of protein import into nucleus	0.048162828
BP	GO:0045416	positive regulation of interleukin-8 biosynthetic process	0.048162828
BP	GO:0070486	leukocyte aggregation	0.048162828
BP	GO:1904590	negative regulation of protein import	4.82E-02
CC	GO:0042611	MHC protein complex	1.33E-18
CC	GO:0071556	integral component of lumenal side of endoplasmic reticulum membrane	3.15E-14
CC	GO:0098553	lumenal side of endoplasmic reticulum membrane	3.15E-14
CC	GO:0042613	MHC class II protein complex	3.15E-14
CC	GO:0009897	external side of plasma membrane	4.65E-12
CC	GO:0062023	collagen-containing extracellular matrix	3.48E-10
CC	GO:0012507	ER to Golgi transport vesicle membrane	4.27E-10
CC	GO:0030666	endocytic vesicle membrane	9.14E-09
CC	GO:0001772	immunological synapse	1.09E-08
CC	GO:0030662	coated vesicle membrane	2.61E-08
CC	GO:0030134	COPII-coated ER to Golgi transport vesicle	3.73E-08
CC	GO:0005774	vacuolar membrane	2.15E-07
CC	GO:0030660	Golgi-associated vesicle membrane	3.16E-07
CC	GO:0030665	clathrin-coated vesicle membrane	3.16E-07
CC	GO:0005765	lysosomal membrane	3.16E-07
CC	GO:0098852	lytic vacuole membrane	3.16E-07
CC	GO:0030176	integral component of endoplasmic reticulum membrane	6.78E-07
CC	GO:0030669	clathrin-coated endocytic vesicle membrane	7.74E-07
CC	GO:0031227	intrinsic component of endoplasmic reticulum membrane	1.12E-06
CC	GO:0030139	endocytic vesicle	3.13E-06
CC	GO:0030135	coated vesicle	8.04E-06

CC	GO:0005770	late endosome	8.42E-06
CC	GO:0045334	clathrin-coated endocytic vesicle	1.08E-05
CC	GO:0005798	Golgi-associated vesicle	2.33E-05
CC	GO:0010008	endosome membrane	2.37E-05
CC	GO:0030136	clathrin-coated vesicle	3.79E-05
CC	GO:0031902	late endosome membrane	5.87E-05
CC	GO:0005775	vacuolar lumen	8.68E-05
CC	GO:0030658	transport vesicle membrane	9.51E-05
CC	GO:0032588	trans-Golgi network membrane	0.000145701
CC	GO:0030667	secretory granule membrane	0.00017274
CC	GO:0043202	lysosomal lumen	0.00017274
CC	GO:0045121	membrane raft	0.000291827
CC	GO:0030670	phagocytic vesicle membrane	0.000291827
CC	GO:0098857	membrane microdomain	0.000291827
CC	GO:0098589	membrane region	0.000423534
CC	GO:0101002	ficolin-1-rich granule	0.000682611
CC	GO:0034774	secretory granule lumen	0.00122006
CC	GO:0045335	phagocytic vesicle	0.001446703
CC	GO:0060205	cytoplasmic vesicle lumen	0.001905705
CC	GO:0031983	vesicle lumen	0.001912319
CC	GO:0030133	transport vesicle	0.002283276
CC	GO:0072562	blood microparticle	0.002706324
CC	GO:0061702	inflammasome complex	0.003168375
CC	GO:0005581	collagen trimer	0.003787943
CC	GO:1904813	ficolin-1-rich granule lumen	0.004533206
CC	GO:0070820	tertiary granule	0.005063595
CC	GO:0005796	Golgi lumen	0.008112787
CC	GO:0005766	primary lysosome	0.014894034
CC	GO:0042582	azurophil granule	0.014894034
CC	GO:0055038	recycling endosome membrane	0.014919751
CC	GO:0005771	multivesicular body	0.015862195
CC	GO:0035577	azurophil granule membrane	0.024692146
CC	GO:0005614	interstitial matrix	0.038562559

CC	GO:0005802	trans-Golgi network	0.039287407
CC	GO:0031901	early endosome membrane	4.38E-02
MF	GO:0008009	chemokine activity	6.21E-13
MF	GO:0042379	chemokine receptor binding	1.27E-12
MF	GO:0042605	peptide antigen binding	1.58E-11
MF	GO:0001664	G protein-coupled receptor binding	1.03E-07
MF	GO:0019955	cytokine binding	1.28E-07
MF	GO:0003823	antigen binding	2.36E-07
MF	GO:0048020	CCR chemokine receptor binding	2.42E-07
MF	GO:0005125	cytokine activity	2.02E-06
MF	GO:0042277	peptide binding	3.71E-06
MF	GO:0019956	chemokine binding	6.93E-06
MF	GO:0005126	cytokine receptor binding	1.02E-05
MF	GO:0019957	C-C chemokine binding	1.81E-05
MF	GO:0004896	cytokine receptor activity	1.81E-05
MF	GO:0023023	MHC protein complex binding	2.18E-05
MF	GO:0033218	amide binding	3.53E-05
MF	GO:0023026	MHC class II protein complex binding	4.00E-05
MF	GO:0048018	receptor ligand activity	4.40E-05
MF	GO:0016493	C-C chemokine receptor activity	0.000253946
MF	GO:0001637	G protein-coupled chemoattractant receptor activity	0.000432623
MF	GO:0004950	chemokine receptor activity	0.000432623
MF	GO:0005201	extracellular matrix structural constituent	0.001270275
MF	GO:0008236	serine-type peptidase activity	0.00302935
MF	GO:0030021	extracellular matrix structural constituent conferring compression resistance	0.00331558
MF	GO:0017171	serine hydrolase activity	0.00331558
MF	GO:0005539	glycosaminoglycan binding	0.004093594
MF	GO:0032395	MHC class II receptor activity	0.004224737
MF	GO:0004252	serine-type endopeptidase activity	0.004422479
MF	GO:0045236	CXCR chemokine receptor binding	0.005005436
MF	GO:0050786	RAGE receptor binding	0.005005436
MF	GO:0005319	lipid transporter activity	0.005005436
MF	GO:0035325	Toll-like receptor binding	0.006370636

MF	GO:0001540	amyloid-beta binding	0.007477871
MF	GO:0001608	G protein-coupled nucleotide receptor activity	0.007477871
MF	GO:0045028	G protein-coupled purinergic nucleotide receptor activity	0.007477871
MF	GO:0070492	oligosaccharide binding	0.011019092
MF	GO:0097153	cysteine-type endopeptidase activity involved in apoptotic process	0.011019092
MF	GO:0005525	GTP binding	0.016737109
MF	GO:0032550	purine ribonucleoside binding	0.017913073
MF	GO:0004175	endopeptidase activity	0.018221873
MF	GO:0001883	purine nucleoside binding	0.018221873
MF	GO:0032549	ribonucleoside binding	0.018221873
MF	GO:0008201	heparin binding	0.01866681
MF	GO:0042287	MHC protein binding	0.01866681
MF	GO:0001882	nucleoside binding	0.019200952
MF	GO:0001614	purinergic nucleotide receptor activity	0.019200952
MF	GO:0008329	signaling pattern recognition receptor activity	0.019200952
MF	GO:0016502	nucleotide receptor activity	0.019200952
MF	GO:0042288	MHC class I protein binding	0.019200952
MF	GO:0043028	cysteine-type endopeptidase regulator activity involved in apoptotic process	0.019200952
MF	GO:0019001	guanyl nucleotide binding	0.019200952
MF	GO:0032561	guanyl ribonucleotide binding	0.019200952
MF	GO:0038187	pattern recognition receptor activity	0.020640282
MF	GO:0051861	glycolipid binding	0.020640282
MF	GO:0015026	coreceptor activity	0.021192721
MF	GO:0035586	purinergic receptor activity	0.033089848
MF	GO:0008083	growth factor activity	0.045817069

BP: Biological Process; MF: Molecular Function; CC: Cellular Component represent

Table S5. The Kyoto Encyclopedia of Genes and Genomes pathway enrichment of 280 common differentially expressed genes with p.adjust < 0.05

ID	Description	p.adjust
hsa04061	Viral protein interaction with cytokine and cytokine receptor	8.82E-16
hsa05150	Staphylococcus aureus infection	5.04E-14
hsa05330	Allograft rejection	1.55E-13
hsa04659	Th17 cell differentiation	2.35E-13
hsa05332	Graft-versus-host disease	3.24E-13
hsa04640	Hematopoietic cell lineage	4.75E-13
hsa04940	Type I diabetes mellitus	4.98E-13
hsa04060	Cytokine-cytokine receptor interaction	2.27E-12
hsa05320	Autoimmune thyroid disease	1.01E-11
hsa04612	Antigen processing and presentation	1.30E-11
hsa04658	Th1 and Th2 cell differentiation	1.33E-11
hsa05310	Asthma	7.06E-11
hsa05323	Rheumatoid arthritis	1.75E-10
hsa05416	Viral myocarditis	7.18E-10
hsa04062	Chemokine signaling pathway	3.52E-09
hsa05322	Systemic lupus erythematosus	4.10E-09
hsa04145	Phagosome	4.10E-09
hsa04672	Intestinal immune network for IgA production	1.21E-08
hsa04514	Cell adhesion molecules (CAMs)	1.70E-08
hsa05321	Inflammatory bowel disease (IBD)	2.06E-08
hsa05169	Epstein-Barr virus infection	4.95E-08
hsa05140	Leishmaniasis	1.41E-07
hsa05166	Human T-cell leukemia virus 1 infection	1.86E-07
hsa04610	Complement and coagulation cascades	4.07E-07
hsa05164	Influenza A	7.80E-07
hsa04064	NF-kappa B signaling pathway	2.93E-06
hsa05145	Toxoplasmosis	7.86E-06
hsa05152	Tuberculosis	4.51E-05
hsa05133	Pertussis	7.54E-05
hsa05142	Chagas disease (American trypanosomiasis)	0.000128259
hsa04621	NOD-like receptor signaling pathway	0.000207421
hsa04668	TNF signaling pathway	0.000272309
hsa05340	Primary immunodeficiency	0.000399749
hsa05168	Herpes simplex virus 1 infection	0.00064826
hsa04660	T cell receptor signaling pathway	0.000772488
hsa04650	Natural killer cell mediated cytotoxicity	0.000910924
hsa05235	PD-L1 expression and PD-1 checkpoint pathway in cancer	0.001334703
hsa05020	Prion diseases	0.002349536
hsa04380	Osteoclast differentiation	0.013895119
hsa04620	Toll-like receptor signaling pathway	0.016455215
hsa05134	Legionellosis	0.02020036

hsa05132	Salmonella infection	0.032470993
hsa05167	Kaposi sarcoma-associated herpesvirus infection	0.036700205
hsa05202	Transcriptional misregulation in cancer	0.036700205
hsa04657	IL-17 signaling pathway	0.036700205
hsa02010	ABC transporters	0.042388998

Table S6. The list of 14 prognostic genes screened from the GSE85047 cohort with p-value < 0.05 in log-rank test.

gene	pvalue	prognostic correlation	regulation in TME
ABCA6	0.000456348	positively	up-regulated
SEPP1	0.000643517	positively	up-regulated
SLAMF8	0.002369878	positively	up-regulated
GPR171	0.006901372	positively	up-regulated
ABCA9	0.007712596	positively	up-regulated
ARHGAP15	0.010944566	positively	up-regulated
IL7R	0.012055187	positively	up-regulated
HLA-DPB1	0.020716568	positively	up-regulated
GZMA	0.023613855	positively	up-regulated
GPR183	0.024085506	positively	up-regulated
CCL19	0.034966337	positively	up-regulated
ITK	0.044202815	positively	up-regulated
FGL2	0.045289209	positively	up-regulated
CD1C	0.047953593	positively	up-regulated

TME: the tumor microenvironment.

Table S7. The list of 56 prognostic genes screened from the GSE49710 cohort with p-value < 0.05 in log-rank test.

gene	pvalue	prognostic correlation	regulation in TME
ABCA6	1.38E-11	positively	up-regulated
SIGLECP3	6.33E-11	positively	up-regulated
LY75	3.80E-10	positively	up-regulated
PTGDS	5.19E-10	positively	up-regulated
SLAMF8	1.44E-09	positively	up-regulated
CD1C	2.70E-09	positively	up-regulated
ABCA9	5.43E-09	positively	up-regulated
CLIC6	1.41E-08	positively	up-regulated
SMOC2	1.97E-08	positively	up-regulated
IRF8	8.78E-08	positively	up-regulated
CTSH	1.11E-07	positively	up-regulated
IL7R	1.44E-07	positively	up-regulated
BIRC3	2.09E-07	positively	up-regulated
GPR171	3.27E-07	positively	up-regulated
CCL19	8.77E-07	positively	up-regulated
RGS1	1.29E-06	positively	up-regulated
FGL2	2.00E-06	positively	up-regulated
UBASH3A	2.10E-06	positively	up-regulated
ITK	2.78E-06	positively	up-regulated

IL32	3.42E-06	positively	up-regulated
CD69	3.83E-06	positively	up-regulated
P2RY14	4.29E-06	positively	up-regulated
FYB	4.36E-06	positively	up-regulated
GPR183	4.64E-06	positively	up-regulated
ZNF683	6.44E-06	positively	up-regulated
HLA-DRB1	8.58E-06	positively	up-regulated
BIN2	1.10E-05	positively	up-regulated
CD48	1.30E-05	positively	up-regulated
HLA-DMB	1.35E-05	positively	up-regulated
ARHGAP15	1.60E-05	positively	up-regulated
HLA-DPB1	2.25E-05	positively	up-regulated
ARHGAP9	2.62E-05	positively	up-regulated
HLA-DMA	3.19E-05	positively	up-regulated
CXCR6	3.83E-05	positively	up-regulated
MOXD1	4.12E-05	positively	up-regulated
CD74	4.38E-05	positively	up-regulated
SEPP1	4.85E-05	positively	up-regulated
TRAC	5.80E-05	positively	up-regulated
TARP	5.84E-05	positively	up-regulated
CD52	6.26E-05	positively	up-regulated
RHOH	7.85E-05	positively	up-regulated

LCK	9.54E-05	positively	up-regulated
CCL5	0.00010068	positively	up-regulated
GZMA	0.000123844	positively	up-regulated
EOMES	0.000166174	positively	up-regulated
APBB1IP	0.000203646	positively	up-regulated
GZMK	0.000244536	positively	up-regulated
GIMAP2	0.000399324	positively	up-regulated
TLR8	0.000510144	positively	up-regulated
CCR2	0.000675186	positively	up-regulated
GPR65	0.000678166	positively	up-regulated
HLA-DRA	0.000809559	positively	up-regulated
SH2D1A	0.002037447	positively	up-regulated
IL33	0.002663504	positively	up-regulated
IGH@	0.02164135	negatively	up-regulated
DPT	0.027918867	negatively	up-regulated

Table S8. The main scripts used in this study.

For ESTIMATE scores:

```
library(limma)
library(estimat
e)
inputFile="forscore.txt"
rt=read.table(inputFile,sep="\t",header=T,check.names=F)
rt=as.matrix(rt)
rownames(rt)=rt[,1]
exp=rt[,2:ncol(
rt)]
dimnames=list(rownames(exp),colnames(exp))
data=matrix(as.numeric(as.matrix(exp)),nrow=nrow(exp),dimnames=dimna
mes)
data=avereps(data)
#group=sapply(strsplit(colnames(data),"\\-"),"[",4)
#group=sapply(strsplit(group,""),"[",1)
#group=gsub("2","1",group)
data=data[,group==0]
out=data[rowMeans(data)>0,]
out=rbind(ID=colnames(out),out)
write.table(out,file="uniq.symbol.txt",sep="\t",quote=F,col.names=F)
filterCommonGenes(input.f="uniq.symbol.txt",
                  output.f="commonGenes.gct",
                  id="GeneSymbol")
estimateScore(input.ds = "commonGenes.gct",
              output.ds="estimateScore.gct",
              platform = c("agilent"))
scores=read.table("estimateScore.gct",skip = 2,header = T)
rownames(scores)=scores[,1]
scores=t(scores[,3:ncol(scores)])
rownames(scores)=gsub("\\.","\\-",rownames(scores))
out=rbind(ID=colnames(scores),scores)
write.table(out,file="scores.txt",sep="\t",quote=F,col.names=F)
```

For Survival analysis:

```
library(survival
)
rt=read.table("E-MTAB-8248_mergedtime_223.txt",header=T,sep="\t",chec
k.names=F)
rt$futime=rt$futime/365
outTab=data.frame()
for(gene in colnames(rt[,4:ncol(rt)])){
```

```
    a=rt[,gene]<=median(rt[,gene])
    diff=survdiff(Surv(futime, fustat) ~a,data = rt)
    pValue=1-pchisq(diff$chisq,df=1)
    outTab=rbind(outTab,cbind(gene=gene,pvalue=pValue))
    pValue=round(pValue,3)
    #pValue=format(pValue, scientific = TRUE)
    fit <- survfit(Surv(futime, fustat) ~ a, data = rt)
    summary(fit)
    pdf(file=paste(gene,".survival.pdf",sep=""),

  width=6,

  height=6)
    plot(fit,
         lwd=2,
         col=c("red","blue"),
         xlab="Time (year)",

  mark.time=T,
         ylab="Survival rate",
         main=paste(gene,"(p=", pValue ,")",sep="") )
    legend("topright",
           c("High","Low"),
           lwd=2,
           col=c("red","blue"))
    dev.off()
  }
  write.table(outTab,file="survival.xls",sep="\t",row.names=F,quote=F)
```

For gene differential expression analysis:

```
  library("limma"
  )
  #setwd("C:\\Users\\M\\Desktop\\um\\10.ImmuneDiff"
  inputFile="forscore.txt"
  scoreFile="scores.txt"
  fdrFilter=0.05
  logFCfilter=1
  score=read.table(scoreFile,sep="\t",header=T,check.names=F)
  #score=score[order(score[,2]),]
  med=median(score[,2])
  conTab=score[score[,2]<med,]
  treatTab=score[score[,2]>=med,]
  con=as.vector(conTab[,1])
  treat=as.vector(treatTab[,1])
  conNum=length(con)
```

```
treatNum=length(treat)
outTab=data.frame()
grade=c(rep(1,conNum),rep(2,treatNum))
rt=read.table(inputFile,sep="\t",header=T,check.names=F)
rt=as.matrix(rt)
rownames(rt)=rt[,1]
exp=rt[,2:ncol(
rt)]
dimnames=list(rownames(exp),colnames(exp))
data=matrix(as.numeric(as.matrix(exp)),nrow=nrow(exp),dimnames=dimna
mes)
data=avereps(data)
data=data[rowMeans(data)>0.1,]
data=cbind(data[,con],data[,treat])
for(i in row.names(data)){
  geneName=unlist(strsplit(i,"\\|",))[1]
  geneName=gsub("\\/", "_", geneName)
  rt=rbind(expression=data[i,],grade=grade)

rt=as.matrix(t(r
t))
  wilcoxTest<-wilcox.test(expression ~ grade, data=rt)
  conGeneMeans=mean(data[i,1:conNum])
  treatGeneMeans=mean(data[i,(conNum+1):ncol(data)])
  logFC=treatGeneMeans-conGeneMeans
  #logFC=log2(treatGeneMeans)-log2(conGeneMeans)
  pvalue=wilcoxTest$p.value
  conMed=median(data[i,1:conNum])
  treatMed=median(data[i,(conNum+1):ncol(data)])
  diffMed=treatMed-conMed
          if( ((logFC>0) & (diffMed>0)) | ((logFC<0) & (diffMed<0)) ){

                outTab=rbind(outTab,cbind(gene=i,conMean=conGeneMe
                ans,treatMean=treatGeneMeans,logFC=logFC,pValue=pv
                alue))
           }
}
pValue=outTab[,"pValue"]
fdr=p.adjust(as.numeric(as.vector(pValue)),method="fdr")
outTab=cbind(outTab,fdr=fdr)
write.table(outTab,file="all.xls",sep="\t",row.names=F,quote=F)
outDiff=outTab[( abs(as.numeric(as.vector(outTab$logFC)))>logFCfilter &
as.numeric(as.vector(outTab$fdr))<fdrFilter),]
write.table(outDiff,file="ImmuneDiff.xls",sep="\t",row.names=F,quote=F)
```

```
up=outTab[( as.numeric(as.vector(outTab$logFC))>logFCfilter &
as.numeric(as.vector(outTab$fdr))<fdrFilter),]
write.table(up,file="ImmuneUp.txt",sep="\t",row.names=F,quote=F)
down=outTab[( as.numeric(as.vector(outTab$logFC))< -logFCfilter &
as.numeric(as.vector(outTab$fdr))<fdrFilter),]
write.table(down,file="ImmuneDown.txt",sep="\t",row.names=F,quote=F)
heatmap=rbind(ID=colnames(data[as.vector(outDiff[,1]),]),data[as.vector(ou
tDiff[,1]),])
write.table(heatmap,file="heatmap.txt",sep="\t",col.names=F,quote=F)
Type=c(rep("low",conNum),rep("high",treatNum))
names(Type)=colnames(data)
Type=as.data.frame(Type)
Type=cbind(ID=rownames(Type),Type)
write.table(Type,file="type.txt",sep="\t",row.names=F,quote=F)
```

For heatmap:

```
rt=read.table("heatmap.txt",sep="\t",header=T,row.names=1,check.names=
F)
#rt=log2(rt+0.1
)
outpdf="heatmap.pdf"
rt=t(scale(t(rt))
)
rt[rt>2]=2
rt[rt< -2] = -2
library(pheatm
ap)
Type=read.table("type.txt",sep="\t",header=T,row.names=1,check.names=
F)
pdf(outpdf,height=15,width=20)
pheatmap(rt, annotation=Type,
         color = colorRampPalette(c("navy", "white", "firebrick3"))(5000),
         cluster_cols = F,
         cluster_row = T,

scale="row",
         #clustering_method = "ward.D2",

fontsize=60,

#order_col =
         #cutree_cols = T,
         show_rownames = F,
         show_colnames = F,
```

```
                fontsize_row=1,
                fontsize_col=3)
dev.off()
```

For GO analysis:

```
library("clusterProfiler")
library("org.Hs.eg.db")
library("enrichplot")
library("ggplot
2")
#setwd("C:\\Users\\M\\Desktop\\um\\14.GO")
rt=read.table("id.txt",sep="\t",header=T,check.names=F)
rt=rt[is.na(rt[,"entrezID"])==F,]
gene=rt$entre
zID
kk <- enrichGO(gene = gene,
                  OrgDb = org.Hs.eg.db,
                  pvalueCutoff =0.05,
                  qvalueCutoff = 1,
                  ont="all",
                  readable =T)
write.table(kk,file="GO.txt",sep="\t",quote=F,row.names = F)
#tiff(file="barplot.tiff",width = 26,height = 20,units
="cm",compression="lzw",bg="white",res=600)
#barplot(kk, drop = TRUE, showCategory =10,split="ONTOLOGY") +
facet_grid(ONTOLOGY~., scale='free')
#dev.off()
tiff(file="dotplot.tiff",width = 20,height = 25,units
="cm",compression="lzw",bg="white",res=1200)
dotplot(kk,showCategory = 10,split="ONTOLOGY") +
facet_grid(ONTOLOGY~., scale='free')
dev.off()
pdf(file="barplot.PDF",width = 8,height = 10)
barplot(kk, drop = TRUE, showCategory =10,split="ONTOLOGY") +
facet_grid(ONTOLOGY~., scale='free')
dev.off()
pdf(file="dotplot.PDF",width = 8,height = 10)
dotplot(kk,showCategory = 10,split="ONTOLOGY") +
facet_grid(ONTOLOGY~., scale='free')
dev.off()
```

For KEGG analysis:

```
library("clusterProfiler")
library("org.Hs.eg.db")
```

```
library("enrichplot")
library("ggplot
2")
#setwd("C:\\Users\\M\\Desktop\\um\\15.KEGG")
rt=read.table("id.txt",sep="\t",header=T,check.names=F)
rt=rt[is.na(rt[,"entrezID"])==F,]
gene=rt$entre
zID
kk <- enrichKEGG(gene = gene, organism = "hsa", pvalueCutoff =0.05,
qvalueCutoff =1)
write.table(kk,file="KEGG.txt",sep="\t",quote=F,row.names = F)
tiff(file="dotplot.tiff",width = 20,height = 25,units
="cm",compression="lzw",bg="white",res=1200)
dotplot(kk, showCategory = 30)
dev.off()
pdf(file="barplotnew.pdf",width = 8,height = 10)
barplot(kk, drop = TRUE, showCategory = 30)
dev.off()
pdf(file="dotplotnew.pdf",width = 8,height = 10)
dotplot(kk, showCategory = 30)
dev.off()
```

For Cox analysis:

```
library(survival
)
library(survmin
er)
#setwd("C:\\Users\\lexb4\\Desktop\\singleGene\\18.multiCox")
rt=read.table("for cox.txt",header=T,sep="\t",check.names=F,row.names=1)
#rt[,"GNPNAT1"]=log2(rt[,"GNPNAT1"]+0)
multiCox=coxph(Surv(futime, fustat) ~ .
                    , data = rt)
multiCoxSum=summary(multiCox)
outTab=data.frame()
outTab=cbind(
coef=signif(multiCoxSum$coefficients[,"coef"], digits=3),
# HR=signif(multiCoxSum$conf.int[,"exp(coef)"], digits=3),
# HR95L=signif(multiCoxSum$conf.int[,"lower .95"], digits=3),
# HR95H=signif(multiCoxSum$conf.int[,"upper .95"], digits=3),
HR.95.CI=paste0(signif(multiCoxSum$conf.int[,"exp(coef)"], digits=3), " (",
signif(multiCoxSum$conf.int[,"lower .95"], digits=3), "-",
signif(multiCoxSum$conf.int[,"upper .95"], digits=3), ")"),
z=signif(multiCoxSum$coefficients[,"z"], digits=3),
pvalue=signif(multiCoxSum$coefficients[,"Pr(>|z|)"], digits=3) )
```

```
#outTab=cbin
d(
#    coef=paste0(coef),
# HR.95.CI=paste0(HR, " (", HR95L, "-", HR95H, ")"),
#
z=paste0(z),
#    pvalue=paste0(pvalue))
outTab=cbind(id=row.names(outTab),outTab)
write.table(outTab,file="2multiCox.xls",sep="\t",row.names=F,quote=F)
```
